# Genomic diversifications of five *Gossypium* allopolyploid species and their impact on cotton improvement

**DOI:** 10.1038/s41588-020-0614-5

**Published:** 2020-04-20

**Authors:** Z. Jeffrey Chen, Avinash Sreedasyam, Atsumi Ando, Qingxin Song, Luis M. De Santiago, Amanda M. Hulse-Kemp, Mingquan Ding, Wenxue Ye, Ryan C. Kirkbride, Jerry Jenkins, Christopher Plott, John Lovell, Yu-Ming Lin, Robert Vaughn, Bo Liu, Sheron Simpson, Brian E. Scheffler, Li Wen, Christopher A. Saski, Corrinne E. Grover, Guanjing Hu, Justin L. Conover, Joseph W. Carlson, Shengqiang Shu, Lori B. Boston, Melissa Williams, Daniel G. Peterson, Keith McGee, Don C. Jones, Jonathan F. Wendel, David M. Stelly, Jane Grimwood, Jeremy Schmutz

**Affiliations:** 1 Department of Molecular Biosciences, The University of Texas at Austin, Austin, TX USA; 2 State Key Laboratory for Crop Genetics and Germplasm Enhancement, Nanjing Agricultural University, Nanjing, China; 3 HudsonAlpha Institute for Biotechnology, Huntsville, AL USA; 4 Department of Soil and Crop Sciences, Texas A&M University System, College Station, TX USA; 5 US Department of Agriculture-Agricultural Research Service, Genomics and Bioinformatics Research Unit, Raleigh, NC USA; 6 College of Agriculture and Food Science, Zhejiang A&F University, Lin’an, China; 7 US Department of Agriculture-Agricultural Research Service, Genomics and Bioinformatics Research Unit, Stoneville, MS USA; 8 Department of Plant and Environmental Sciences, Clemson University, Clemson, SC USA; 9 Department of Ecology, Evolution, and Organismal Biology, Iowa State University, Ames, IA USA; 10 The US Department of Energy Joint Genome Institute, Walnut Creek, CA USA; 11 Institute for Genomics, Biocomputing and Biotechnology and Department of Plant and Soil Sciences, Mississippi State University, Mississippi State, MS USA; 12 School of Agriculture and Applied Sciences, Alcorn State University, Lorman, MS USA; 13 Agriculture and Environmental Research, Cotton Incorporated, Cary, NC USA

**Keywords:** Plant hybridization, Epigenomics, Sequence annotation, Genome assembly algorithms, Plant breeding

## Abstract

Polyploidy is an evolutionary innovation for many animals and all flowering plants, but its impact on selection and domestication remains elusive. Here we analyze genome evolution and diversification for all five allopolyploid cotton species, including economically important Upland and Pima cottons. Although these polyploid genomes are conserved in gene content and synteny, they have diversified by subgenomic transposon exchanges that equilibrate genome size, evolutionary rate heterogeneities and positive selection between homoeologs within and among lineages. These differential evolutionary trajectories are accompanied by gene-family diversification and homoeolog expression divergence among polyploid lineages. Selection and domestication drive parallel gene expression similarities in fibers of two cultivated cottons, involving coexpression networks and *N*^6^-methyladenosine RNA modifications. Furthermore, polyploidy induces recombination suppression, which correlates with altered epigenetic landscapes and can be overcome by wild introgression. These genomic insights will empower efforts to manipulate genetic recombination and modify epigenetic landscapes and target genes for crop improvement.

## Abstract (web summary)

Sequencing and genomic diversification of five allopolyploid cotton species provide insights into polyploid genome evolution and epigenetic landscapes for cotton improvement.

## Main

**Fig. 1:**
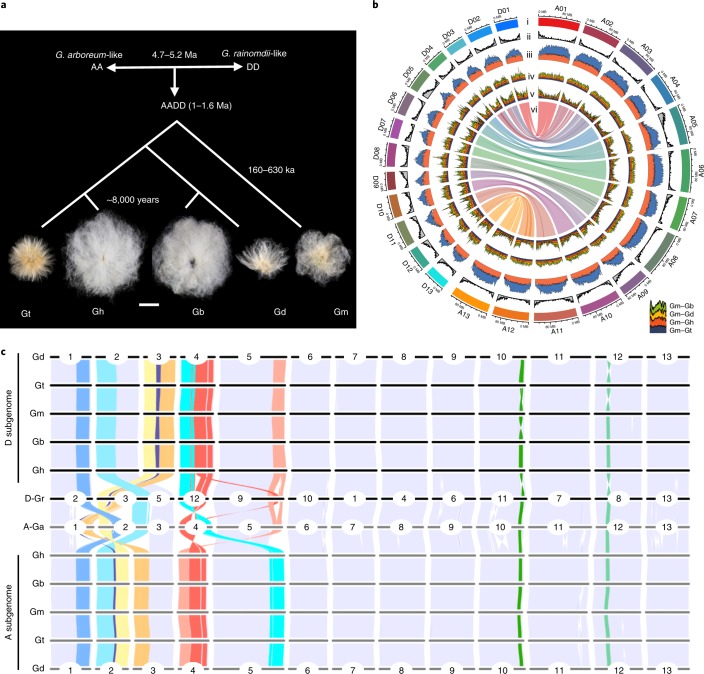
Sequencing features of five cotton allotetraploid species. **a**, Evolution and domestication of five polyploid lineages, Gh, Gb, Gt, Gd and Gm, after polyploidization between an A-genome African species (Ga-like) and a D-genome American species (Gr-like). Typical seeds from each species are shown. The divergence time estimates are based on 21,567 single orthologs among the 5 species by using the synonymous substitution rate (*r*) of 3.48 × 10^−9^ (Methods and Supplementary Note). Scale bar, 10 mm; ka, thousand years ago. **b**, Chromosomal features and synteny of the Gm genome. Notes in circos plots: (i) estimated lengths of 13 A and 13 D homoeologous pseudochromosomes; (ii) distribution of annotated genes; (iii) TE content (*Gypsy*, steel blue; *Copia*, grey; other repeats, orange); (iv,v) stacked SNP (iv) and indel (v) densities between Gm and Gb, Gd, Gh and Gt, respectively (see inset), and (vi) syntenic blocks between the homoeologous A and D chromosomes. The densities in plots in (ii)–(v) are represented in 1 Mb with overlapping 200-kb sliding windows. **c**, Genome-wide syntenic relationships among A and D subgenomes in five allotetraploids relative to the A-genome-like Ga (A_2_ genome) and D-genome-like Gr (D_5_ genome). Structural variations among syntenic blocks are marked with colored ribbons. Source data

Polyploidy or whole-genome duplication provides genomic opportunities for evolutionary innovations in many animal groups and all flowering plants^1–5^, including most important crops such as wheat, cotton and canola or oilseed rape^6–8^. The common occurrence of polyploidy may suggest its advantage and potential for selection and adaptation^2,3,9^, through rapid genetic and genomic changes as observed in newly formed *Brassica napus*^10^, *Tragopogon miscellus*^11^ and polyploid wheat^12^, and/or largely epigenetic modifications as in *Arabidopsis* and cotton polyploids^5,13^. Cotton is a powerful model for revealing genomic insights into polyploidy^3^, providing a phylogenetically defined framework of polyploidization (~1.5 million years ago (Ma))^14^, followed by natural diversification and crop domestication^15^. The evolutionary history of the polyploid cotton clade is longer than that of some other allopolyploids, such as hexaploid wheat (~8,000 years)^12^, tetraploid canola (~7,500 years)^16^ and tetraploid *Tragopogon* (~150 years)^11^. Polyploidization between an A-genome African species (*Gossypium arboreum* (Ga)-like) and a D-genome American species (*G. raimondii* (Gr)-like) in the New World created a new allotetraploid or amphidiploid (AD-genome) cotton clade (Fig. 1a)^14^, which has diversified into five polyploid lineages, *G. hirsutum* (Gh) (AD)_1_, *G. barbadense* (Gb) (AD)_2_, *G. tomentosum* (Gt) (AD)_3_, *G. mustelinum* (Gm) (AD)_4_ and *G. darwinii* (Gd) (AD)_5_. *G. ekmanianum* and *G. stephensii* are recently characterized and closely related to Gh^17^. Gh and Gb were separately domesticated from perennial shrubs to become annualized Upland and Pima cottons^15^. To date, global cotton production provides income for ~100 million families across ~150 countries, with an annual economic impact of ~US$500 billion worldwide^6^. However, cotton supply is reduced due to aridification, climate change and pest emergence. Future improvements in cotton and sustainability will involve use of the genomic resources and gene-editing tools becoming available in many crops^9,18,19^.

Cotton genomes have been sequenced for the D-genome (Gr)^20^ and A-genome (Ga)^21^ diploids and two cultivated tetraploids^22–26^. These analyses have shown structural, genetic and gene expression variation related to fiber traits and stress responses in cultivated cottons, but the impact of polyploidy on selection and domestication among the wild and cultivated polyploid cotton species remains poorly understood^6^. Here we report high-quality genomes for all five allotetraploid species and show that despite wide geographic distribution and diversification, allotetraploid cotton genomes retained the syntenic gene content and genomic diversity relative to respective extant diploids. Evolutionary rate heterogeneities, gene loss and positively selected genes characterize the two subgenomes of each species but differ among polyploid lineages. Transposable elements (TEs) are dynamically exchanged between the two subgenomes, facilitating genome-size equilibration following allopolyploidy. Gene expression diversity in the fiber tissues involves selection, coexpression networks and *N*^6^-methyladenosine (m^6^A) RNA modifications. In cultivated polyploid cottons, recombination suppression correlates with DNA hypermethylation and weak chromatin interactions and can be overcome by wild introgression and possibly epigenetic remodeling. The results offer unique insights into polyploid genome evolution and provide valuable genomic resources for cotton research and improvement.

## Results

### Sequencing, assembly and annotation

**Extended Data Fig. 1:**
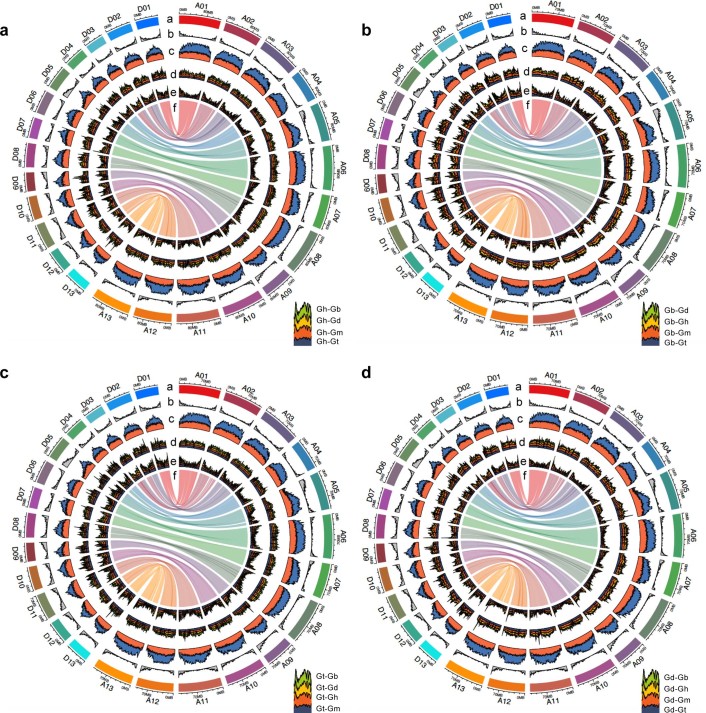
Sequencing features of four cotton allotetraploid species. **a**–**d**, Chromosomal features and synteny of *G. hirsutum* (Gh) (**a**), *G. barbadense* (Gb) (**b**), *G. tomentosum* (Gt) (**c**), and *G. darwinii* (Gd) (**d**) genomes. Notes in the circos plots: (a) estimated lengths of 13 A and 13 D homoeologous pseudochromosomes; (b) density distribution of annotated genes; (c) TE content (*Gypsy*, steel blue; *Copia*, grey; other repeats, orange); (d, e) stacked SNP (d) and INDEL (e) densities between species, respectively (see inset); (f) syntenic blocks between the homoeologous A and D chromosomes. The densities in plots in (b-e) are represented in 1 Mb with overlapping 200-kb sliding windows. Source data

Sequencing of the five allotetraploid cotton genomes entailed using complementary whole-genome shotgun strategies, including sequencing by single-molecule real-time (PacBio SEQUEL and RSII, ~440× genome equivalent), Illumina (HiSeq and NovaSeq, ~286×) (Supplementary Dataset 1a) and chromatin conformation capture (Hi-C seq) (~326×) (Methods). Homozygous single nucleotide polymorphisms (SNPs) and insertions/deletions (indels) were also used to correct the consensus sequence (Supplementary Dataset 1b,c). The rate of anchored scaffolds is 97% in Gb and 99% or higher in the other 4 species. Scaffolds were oriented, ordered and assembled into 26 pseudo-chromosomes with very low (0.1–0.8%) gaps (Table 1 and Supplementary Dataset 1d). The assembled genomes range in size from 2.2 to 2.3 gigabase pairs (Gbp; Table 1), slightly smaller than the sum of the two A- and D-genome diploids (1.7/A + 0.8/D ≈ 2.5 Gbp/AD)^20,21^. Nearly 73% of the assembled genomes are repeats and TEs (Supplementary Dataset 1e), predominantly in pericentromeric regions in Gm (Fig. 1b) and the other 4 species (Extended Data Fig. 1). The completeness and contiguity of these genomes compare favorably with Sanger-based sequences of sorghum^27^ and *Brachypodium*^28^.

**Table 1:** Genome assembly and annotation statistics for five allotetraploid cotton species

Genomic features	Gh	Gb	Gm	Gt	Gd
Estimate of genome size (bp)	2,305,241,538	2,195,804,943	2,315,094,184	2,193,557,323	2,182,957,963
Number of scaffolds	1,025	2,048	383	319	334
Total length of scaffolds (Mb)	2,305.2	2,195.8	2,315.1	2,193.6	2,183.0
Scaffold N50L (Mb)	108.1	93.8	106.8	102.9	101.9
Number of contigs	6,733	4,766	2,147	750	821
Total length of contigs (Mb) and gap (%)^a^	2,302.3 (0.1%)	2,193.9 (0.1%)	2,297.5 (0.8%)	2,189.2 (0.2%)	2,178.1 (0.2%)
Contig N50L (Mb)	0.7839	1.8	2.3	10	9.1
Genome in chromosomes (%)	98.9	97.0	99.0	99.2	99.1
Number of genes	75,376	74,561	74,699	78,338	78,303
Repeat sequences (%)	73.21	72.24	72.85	72.24	72.29

^a^A gap is a representation of the assembled sequence with unknown sequence information. bp, base pair; Mb, megabase pairs.

The euchromatic sequences of 5 polyploid genomes are complete (Supplementary Note), as supported by BUSCO scores (>97%) and 36,880 (>99%) primary transcripts from the Gr version 2 release^20^ (Supplementary Dataset 1b), with the number of protein-coding genes predicted to range from 74,561 (Gb) to 78,338 (Gt; Table 1), which are 3,000–4,000 more than reported in Gh and Gb^23^. Although the A subgenome (1.7 Gbp) is twice the size of the D subgenome (0.8 Gbp)^20,21^, mirroring the ancestral state of their extant diploids, the two have similar numbers of protein-coding genes (ratio of D/A ≈ 1.06; Supplementary Dataset 1f).

As an indication of the improved contiguity (Supplementary Note), the contig length in the Gh genome increases 6.9-fold with a 7.7-fold reduction in fragmentation (6,733 versus 51,849), compared to the published sequences^22^. The improvement is substantial in the Gb genome with a 15.9-fold reduction in N50 contigs and a 23-fold increase in N50 contig length (from 77.6 to 1,800 kilobase pairs (kb)). Moreover, most quality scores are 2-5-fold higher in the 3 wild polyploid species than in Gh and Gb (Table 1).

**Extended Data Fig. 2:**
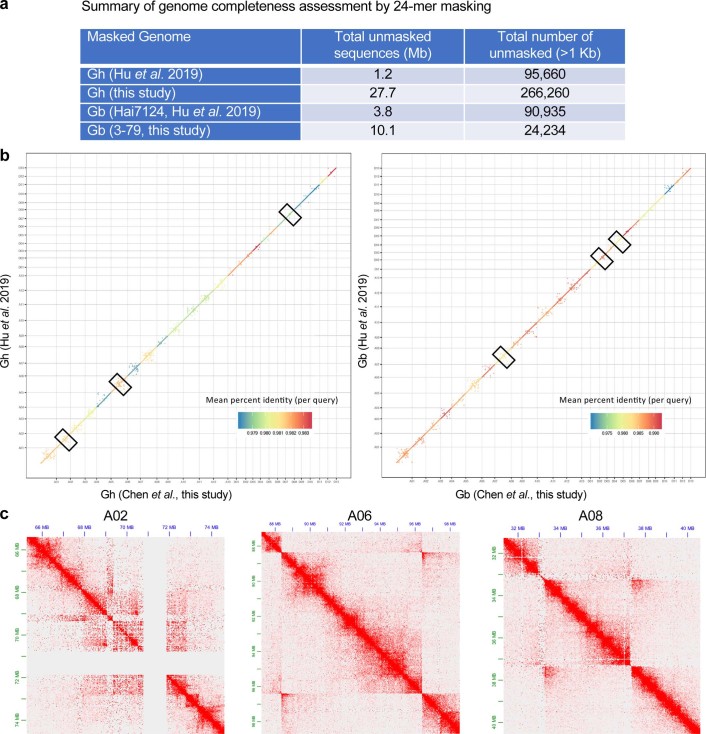
Summary of completeness assessment and collinearity and similarity between *G. hirsutum* (Gh) and *G. barbadense* (Gb) genomes. **a**, Summary of genome completeness assessment by 24-mer reciprocal masking between the published^22^ and our assemblies of Gh and Gb genomes. **b**, Nucleotide alignment dot plots comparing the collinearity and similarity between the genomes of Gh (published^22^ vs. this study, left panel) and Gb (Hai7124^22^ vs. 3-79 of this study, right panel). Plots show *y* axis (bottom to top) for chromosomes A01-A13 and D01-D13^22^ and *x* axis (left to tight) for chromosomes A01-13 and D01-D13 (this study). Boxed regions represent inversions and rearrangements assessed using Hi-C data. Minimum nucleotide alignment length = 1 Kb; color scale, mean percent identity per query. **c**, Hi-C interaction maps indicating rearrangements and inversions in the published Gh genome^22^ with several small rearrangements flanking a large 200-Kb gap in A02, a large inversion in A06, and rearrangements in D08. Source data

Reciprocal 24-nucleotide masking and syntenic analyses show that our Gh and Gb assemblies have ~23- and 2.7-fold more unique sequences, respectively, than the published ones^22^ also with variable gap sizes (10–200 kb; Extended Data Fig. 2a). Some specific genes are present in our annotations and the published data, which are largely related to gene copy number variation (more decreases than increases). Other differences include inversions (132–133 megabase pairs (Mb)) with two large ones (A06 and D03) present in similar regions of both Gh and Gb^22^ (Extended Data Fig. 2b), which could result from errors and/or unresolved alternative haplotypes; these inversions were confirmed using Hi-C data (Extended Data Fig. 2c). Notably, the published Hai7124 strain^22^ is a Gb local strain that is different from Gb 3-79, and Gh TM-1 strains may vary; these can also contribute to the observed variation.

### Evolution within and between five polyploids

**Extended Data Fig. 3:**
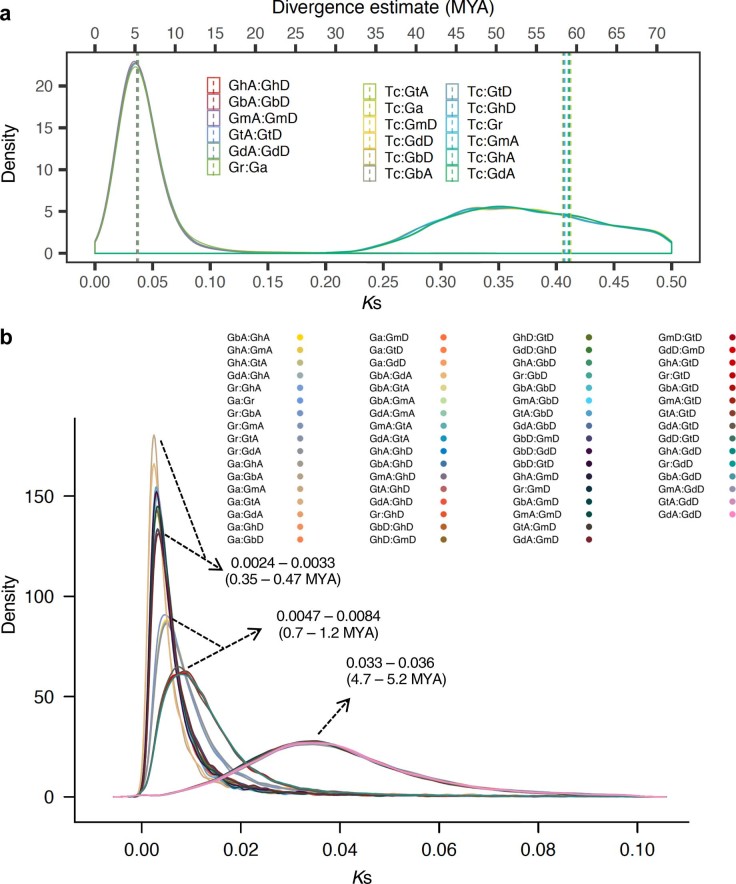
Estimates of divergence time based on synonymous substitution rates (*Ks*). **a**, The divergence time is estimated to be 58-59 million years ago (Mya) between *Theobroma cacao* and *Gossypium*. Data shown using *K*s bin size of 0.001. Divergence time [T = *Ks*/(2*r*)] was estimated using the synonymous substitution rate (*r*) of 3.48 × 10^−9^ synonymous substitutions per synonymous site per year^17^ and 10,562 single copy orthologs between subgenomes and species. *K*s values >1 were removed to eliminate saturated synonymous sites. **b**, The synonymous substitution rate, *K*s, distribution for orthologs (n = 21,567), and estimates of divergence time between allotetraploid subgenomes and progenitor-like diploid genomes. Gh: *G. hirsutum*; Gb: *G. barbadense*; Ga: *G. arboreum*; Gr: *G. raimondii*; Gm: *G. mustelium*. Using a penalized-likelihood based on the concatenated nuclear tree (including branch lengths), the divergence between diploid-tetraploid clade is estimated to be 1-1.6 Mya. Source data

**Extended Data Fig. 4:**
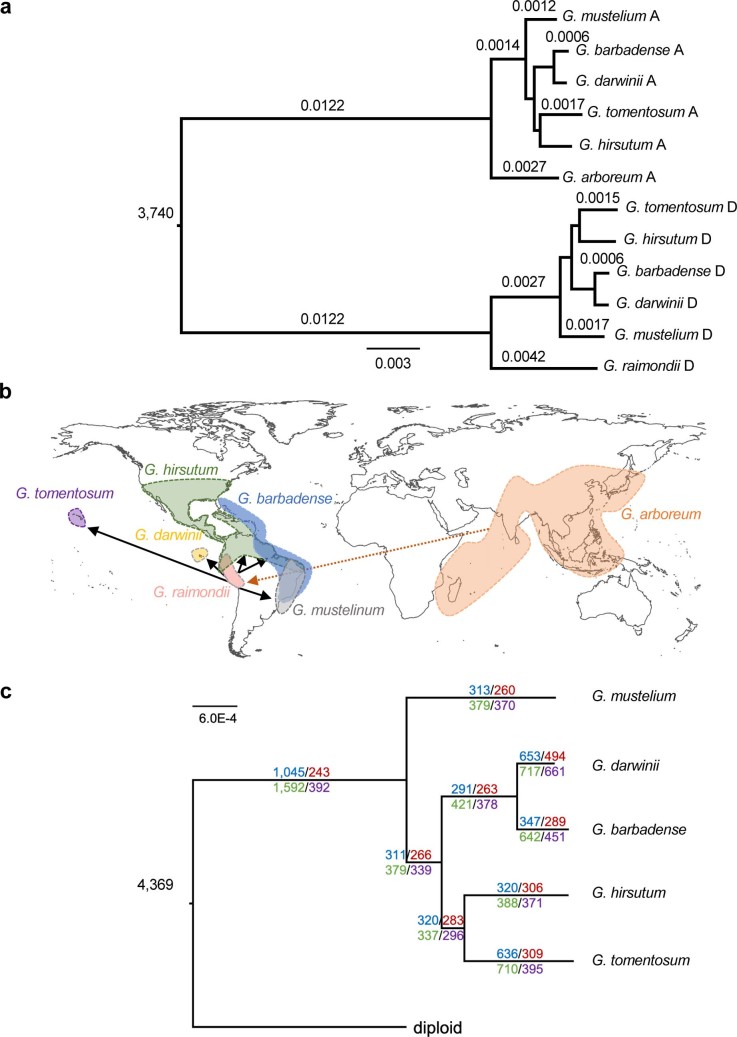
Monophyletic origin and diversification of five allotetraploid species. **a**, The phylogeny of the polyploid species using 18,672 orthologous (37,344 homoeologous) genes and improved coalescence analysis. **b**, Geographic distribution and diversification of the five allotetraploid species *G. hirsutum*, *G. barbadense*, *G. tomentosum*, *G. darwinii*, and *G. mustelium* and their progenitor-like diploids, *G. arboreum* and *G. raimondii*. The world map was made using R scripts, and the distribution maps were redrawn based on published maps for Gd, Gt, and Gm^30^, Gh and Gb^31^, and diploid cultivated cottons^32^. **c**, Patterns of gene gain and loss using 4,369 single-copy orthologs (SCOs) (out of total 32,622), which are present in both diploids and in one or more allotetraploids. Numbers above and below each branch indicate number of gene gain (A-blue/D-red subgenome) or loss (A-green/D-purple subgenome), respectively. Source data

Using the diploid^20,21^ and 5 polyploid cotton genomes, we estimated divergence at 58–59 Ma between *Gossypium* and its relative *Theobroma cacao* (Extended Data Fig. 3a and Supplementary Note), 4.7–5.2 Ma between the extant diploids (Extended Data Fig. 3b), and 1.0–1.6 Ma between polyploid and diploid clades. Genome-wide phylogenetic analysis (Extended Data Fig. 4a) supports a monophyletic origin for the five allotetraploid species^29^. Within the polyploid clade, the highest divergence (~0.63 Ma) occurs between Gm and the other 4 species, with the most recent divergence (~0.20 Ma) between Gb and Gd. This genomic diversification was accompanied by biogeographic radiation to the Galapagos Islands (Gd), the Hawaiian Islands (Gt), South America (northeastern Brazil) (Gm)^30^, Central and South America, the Caribbean, and the Pacific (Gh and Gb)^31^, with separate distribution and domestication of diploid cultivated cottons in southern Arabia, North Africa, western India and China^32^ (Extended Data Fig. 4b). Over the last 8,000 years, Upland (Gh) and Pima (Gb) cottons were independently domesticated in northwest South America and the Yucatan Peninsula of Mexico, respectively, under strong human selection, leading to the modern annualized crops^15^.

After whole-genome duplication, duplicate genes may be lost or diverge in functions^33^, but the pace of this process has rarely been studied in allopolyploids. Using 17,136 homoeolog pairs shared among all 5 allotetraploid species, we demonstrate that most (14,583, 85.5%) homoeolog pairs evolved at statistically indistinguishable rates throughout the polyploid clade relative to the diploids (Supplementary Dataset 2a), but those with rate shifts occur more commonly in the A (1,476, 8.5%) than in the D (845, 5%) subgenome. We further revealed that the D homoeologs generally acquire substitution mutations more quickly than the A homoeologs in most lineages, whereas the Gh and Gt lineages experience a greater rate of divergence in the A than in the D homoeologs (Supplementary Dataset 2b). This relative acceleration of A-homoeolog divergence is mirrored in lineage-specific rate tests; the Gh/Gt clade including Upland cotton has the fastest evolving A homoeologs and the slowest evolving D homoeologs among five polyploids. These results demonstrate pervasive lineage-specific rate heterogeneities between subgenomes and among different polyploid cottons.

We examined patterns of gene loss and gain using 4,369 single-copy orthologs (SCOs), which are present in both diploids and in one or more allotetraploids (Extended Data Fig. 4c). Analysis of gene loss and gain among these basally shared homoeologs in the five polyploid lineages showed the highest level of net gene loss between the initial polyploidization and Gm, with threefold higher levels in the A subgenome (547 net gene losses) than in the D subgenome (149). Other polyploids have fewer gene losses with no subgenomic bias.

**Fig. 2:**
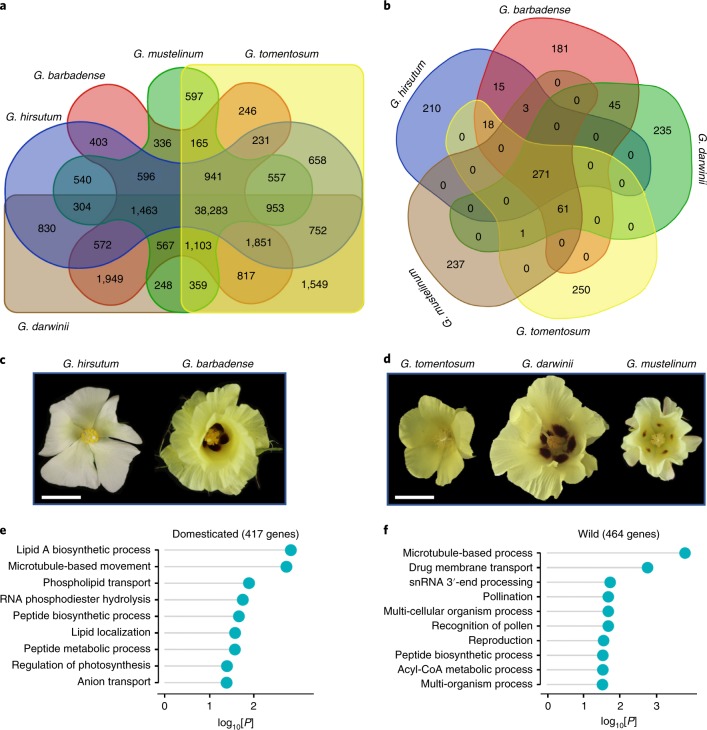
Gene family expansion and contraction in cultivated and wild allotetraploid cotton species. **a**, Venn diagram representing the shared orthologous groups (orthogroups) between cotton species. No species-specific orthogroups were identified by Orthofinder (Methods). **b**, Venn diagram of R-gene family expansion and contraction in these five species. **c**,**d**, Flower morphology of two cultivated (**c**) and three wild (**d**) polyploid cotton species. Scale bars, 30 mm. **e**,**f**, Gene Ontology (GO)-term enrichment of the shared genes in polyploid domesticated cottons (**e**) and wild species (**f**). Source data

Among the homoeologs shared by all five polyploid species (Fig. 2a), the number of genes under positive selection (*K*_a_/*K*_s_ values > 1) is the highest (3,200–3,300) in Gm with the longest branch relative to others, and the lowest between Gb and Gd (~1,100), the most recently diverged polyploid clade (Supplementary Dataset 3). Across different polyploid lineages, 10–20% more D homoeologs are under positive selection than A homoeologs, suggesting a concerted evolutionary impact on subgenomic functions in all polyploid species.

### Genomic diversity among five polyploids

The two subgenomes in each of the five polyploid species are highly conserved at the chromosomal, gene content and nucleotide levels (Fig. 1b and Extended Data Fig. 1). The D subgenomes have fewer and smaller inversions than the A subgenomes (Fig. 1c), as reported for Gh^25^, except for a few small inversions in D10 of Gt–Gm and Gm–Gb and D12 of Gd–Gt–Gm. This level of structural conservation is similar to some polyploids such as wheat^7^ and *Arabidopsis suecica*^34^, but is different from others such as *B. napus*^10^, peanut^35^ and *T. miscellus*^11^, which show rapid homoeologous shuffling.

The genomic conservation is extended to gene order, collinearity and synteny (Fig. 1c). Among the annotated genes (74,561–78,338), 56,870 orthologous groups or 65,300 genes (32,650 homoeologous pairs) (84–88%) are shared among all 5 species (Fig. 2a and Supplementary Dataset 1f).

The number of SNPs is in the range of 4–12 million (1.7–5.2 SNPs kb^−1^) or 0.19–0.53% among 5 polyploid genomes (Supplementary Dataset 4 and Supplementary Note). Gm has the highest SNP level (0.53%) relative to the other 4 species, with the lowest between the most recently diverged species Gb and Gd (~0.19%). Similar trends of indels range from ~5.55 Mb (~0.76%) in Gm–Gt to ~3.35 Mb (~0.34%) in Gb–Gd (Extended Data Fig. 1 and Supplementary Dataset 5). The level of overall variation of SNPs and indels among cotton species is low, comparable to natural variation (3.5–4.1 SNPs kb^−1^) between *Brachypodium* accessions^28^ but lower than that (~7.4 SNPs kb^−1^) for subspecies of rice^36^. SNPs are more frequent in pericentromeric regions, while indel distributions coincide with gene densities (Fig. 1b and Extended Data Fig. 1).

### TE exchanges between two subgenomes that equilibrate the genome-size variation

**Fig. 3:**
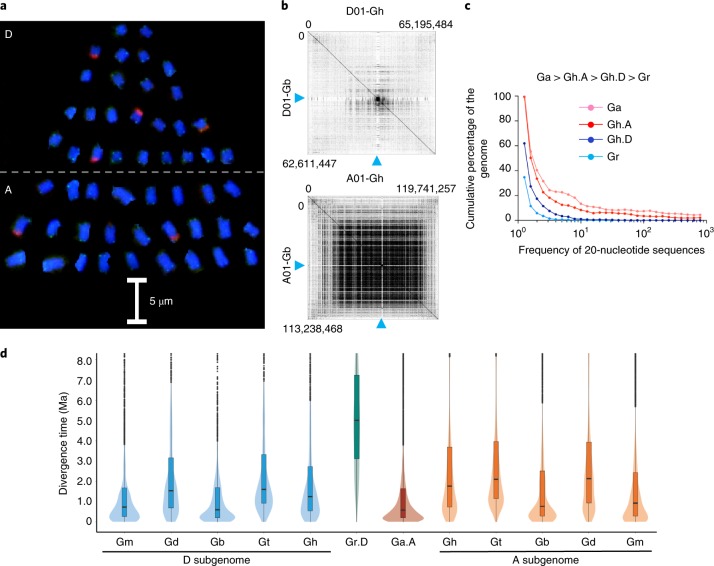
Genomic diversification of A and D subgenomes in five allotetraploid cotton species. **a**, Chromosome painting of Gh using DNA probes to label telomeres (green) and 25S ribosomal DNA (red); DNA is stained by 4′,6-diamidino-2-phenylindole (DAPI, blue). The A (lower half) and D (upper half) homoeologous chromosomes are separated and rearranged into a tree shape. **b**, Pairwise comparison (dot plots) of 18-nucleotide sequences between the Gh and Gb homoeologous chromosomes D01 (top) and A01 (bottom) using Genome Pair Rapid Dotter (Gepard) plot analysis (Methods). The blue arrowheads indicate approximate centromeric locations. Genomic length positions are shown in each plot. **c**, Cumulative percentages (*y* axis) of 20-nucleotide sequences and their frequencies (*x* axis) in the A and D subgenomes of Gh relative to Ga (A) and Gr (D). **d**, The divergence time (Ma) of TEs (*Copia* and *Gypsy*) in the A and D subgenomes relative to their A- and D-genome-like diploids, Ga.A and Gr.D, respectively. The divergence time was estimated using the synonymous substitution rate (*r*) of 3.48 × 10^−9^ (Methods and Supplementary Note). Source data

**Extended Data Fig. 5:**
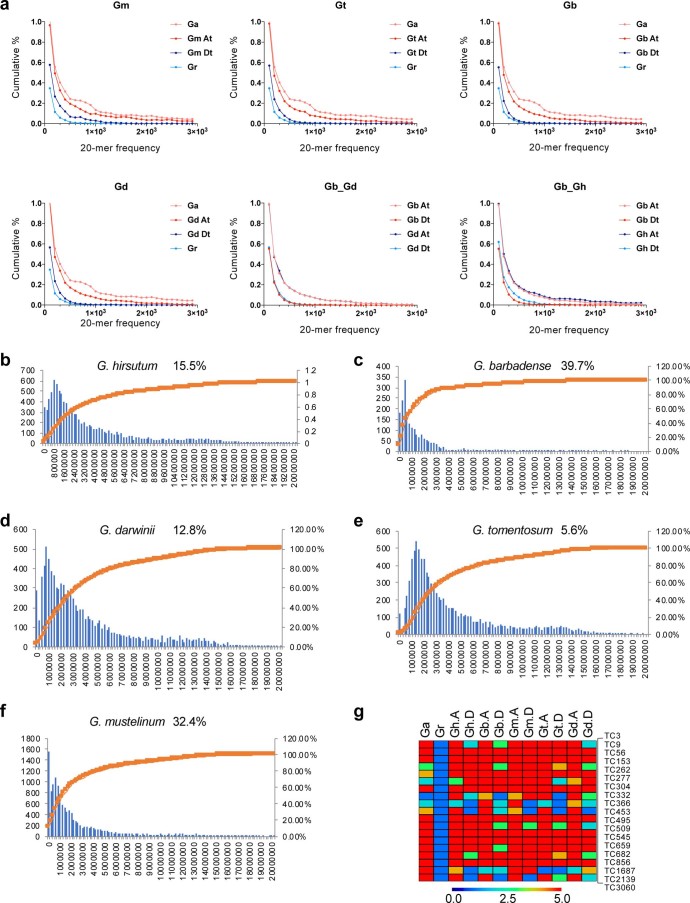
Analysis of 20-nucleotide sequence distributions in subgenomes and *Copia* and *Gypsy* insertion time in five allotetraploid cotton species. **a**, Cumulative percentage (*y* axis) of 20-nucleotide sequences and their frequencies (*x* axis) is lower in the A subgenome than in the A (Ga) genome and higher in the D subgenome than in the D (Gr) genome in *G. mustelium* (Gm), *G. tomentosum* (Gt), *G. barbadense* (Gb), and *G. darwinii* (Gd) (from left to right). **b**-**f**, Number of *Copia* and *Gypsy* elements (*y* axis, left) relative to the estimated time of insertion (*x* axis) in *G. hirsutum* (**b**), *G. barbadense* (**c**), *G. darwinii* (**d**), *G. tomentosum* (**e**), and *G. mustelinum* (**f**). The right (*y* axis) shows cumulative % of *Copia* and *Gypsy* in the genome over divergence time (orange line). The number shown in each species indicates cumulative % of *Copia* and *Gypsy* at ~600 Kya. Note: Divergence time [T = *Ks*/(2*r*)] was estimated using the synonymous substitution rate (*r*) of 3.4 × 10^−9^ synonymous substitutions per synonymous site per year. **g**, Movement of TEs from the A subgenomes to the D subgenomes in allotetraploids. The number of each TE cluster (TC3-TC3060, top-bottom) is shown in the right. Color scale, TE density. Source data

The size difference between the Ga (~1.7 Gbp) and Gr (~0.8 Gbp)^20,21^ genomes is preserved in the respective A and D subgenomes of the 5 allotetraploid species (Fig. 3a). The A subgenome consists of a substantial amount of repetitive DNA in centromeric and pericentromeric regions (Fig. 3b). However, the A subgenome has 4.0–5.9% lower repetitive DNA content than the A-genome diploid (Ga), whereas the D subgenome has 1.5–2.9% higher content than the D-genome diploid (Gr) in Gh (Fig. 3c) and the other 4 species (Extended Data Fig. 5a). Consistently, the D subgenome has 10–20% more long terminal repeat (LTR) TEs than the D-genome diploid, while the A subgenome has 3–11% fewer LTRs than the A-genome diploid. These changes in subgenomic TEs may account for slight genome downsizing (Table 1) and genome-size equilibration following allopolyploidy in all five species, suggesting that the ‘evolutionary tape’ is replayed across polyploid lineages.

*Copia-* and *Gypsy*-like TEs are the most abundant LTRs in the Gh genome^25^. Estimates indicate that divergence of 5.6% (Gt) to 15.5% (Gh) and 39.7% (Gb) LTRs occurred during polyploid diversification (<0.6 Ma; Extended Data Fig. 5b–f). Since polyploid formation, LTRs increased substantially in the D subgenome of all five polyploids (Fig. 3d). The results indicate activation of LTRs in the D subgenome following polyploidization or movement of LTRs from the A to D subgenome^37^. Indeed, some *Copia-* and *Gypsy*-like elements are present in the D subgenome but absent in the extant D-genome diploid (Extended Data Fig. 5g).

### Gene family diversification

The domesticated (Gh and Gb) and wild (Gm, Gt and Gd) cotton species share 417 (403) and 464 (359) unique genes (orthogroups) in respective groups (Fig. 2a), and no species-specific orthogroups are identified, although they possess distinct phenotypic traits such as fiber length (Fig. 1a) and flower morphology (Fig. 2c,d). The unique genes in the two domesticated cottons are over-represented in biological processes such as microtubule-based movement and lipid biosynthetic process and transport in the domesticated cottons (Fig. 2e; *P* < 0.05), reflecting the traits related to fiber development and cottonseed oil. Moreover, many of these genes are under positive selection and overlap regions of domestication traits including fiber yield and quality in Upland cotton^38^ (Supplementary Dataset 6). The unique genes in all three wild polyploid species, however, are enriched for pollination and reproduction (Fig. 2f), suggesting a role of these genes in reproductive adaptation in natural environments.

Plants have evolved an intricate innate immune system to protect them from pathogens and pests through intracellular disease-resistance (R) proteins as a defense response^39^. Among the R genes (Methods and Supplementary Note), each species has its unique R genes with very few genes shared between species (Fig. 2b and Supplementary Dataset 7), despite 5 wild and cultivated species sharing a core R-gene set (271), suggesting extensive diversification of R genes during selection and domestication. This is in contrast to a shared set of unique genes (related to fiber and seed traits) between the two cultivated species and the other shared set (related to reproductive and adaptive traits) among the three wild species (Fig. 2a)

Between the two subgenomes, the D subgenome has higher numbers of R genes (7.8%) than does the A subgenome (*P* = 0.0126, Student’s *t*-test; Supplementary Dataset 7). Using the published data^40^, we found expression induction of ~96% of 291 and 384 predicted R genes in the A and D subgenomes, respectively, by bacterial blight pathogens; 19 in D and 7 in A are upregulated at significant levels (error corrected, FDR = 0.05 and *P* < 0.001, exact test), while a similar trend of R-gene expression is observed after the reniform nematode attack (Supplementary Dataset 8), suggesting a contribution of the D-genome species to disease-resistance traits.

### Gene expression diversity

**Extended Data Fig. 6:**
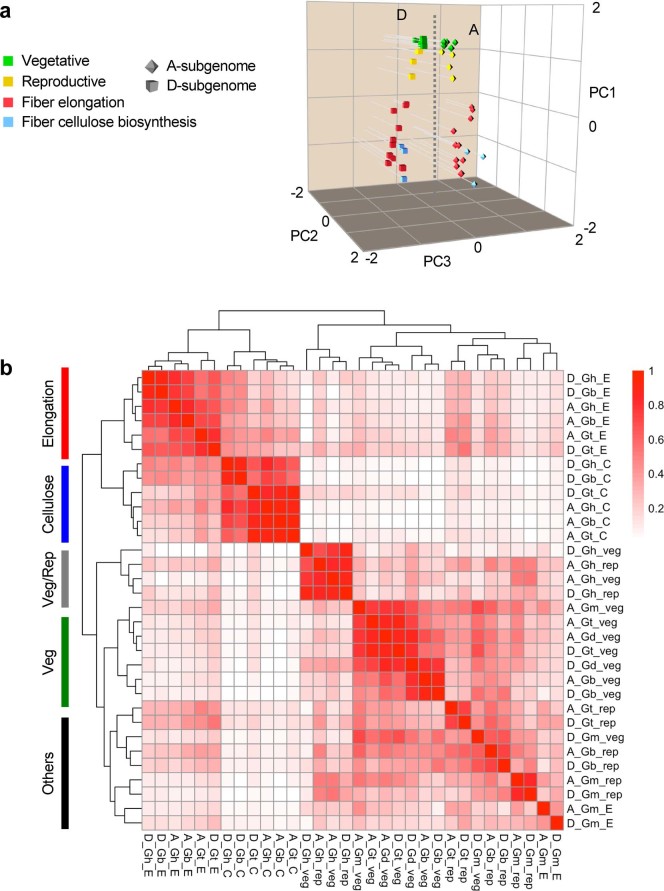
Gene expression diversity between subgenomes and among different developmental stages and five allotetraploid cotton species. **a**, Principal component analysis (PCA) of all genes during vegetative (leaf, stem, and root), reproductive (ovules at 0-35 DAP and square), fiber elongation (7, 14, and 21 DAP), and cellulose biosynthesis (28 and 35 DAP) stages, separating gene expression diversity among different developmental stages and between A and D subgenomes (marked by the dotted lines. **b**, Clustering analysis of 96 RNA-seq datasets with 2 biological replicates in fiber elongation (E), cellulose biosynthesis (C), vegetative (veg), and reproductive (rep) stages of cotton development. Source data

**Extended Data Fig. 7:**
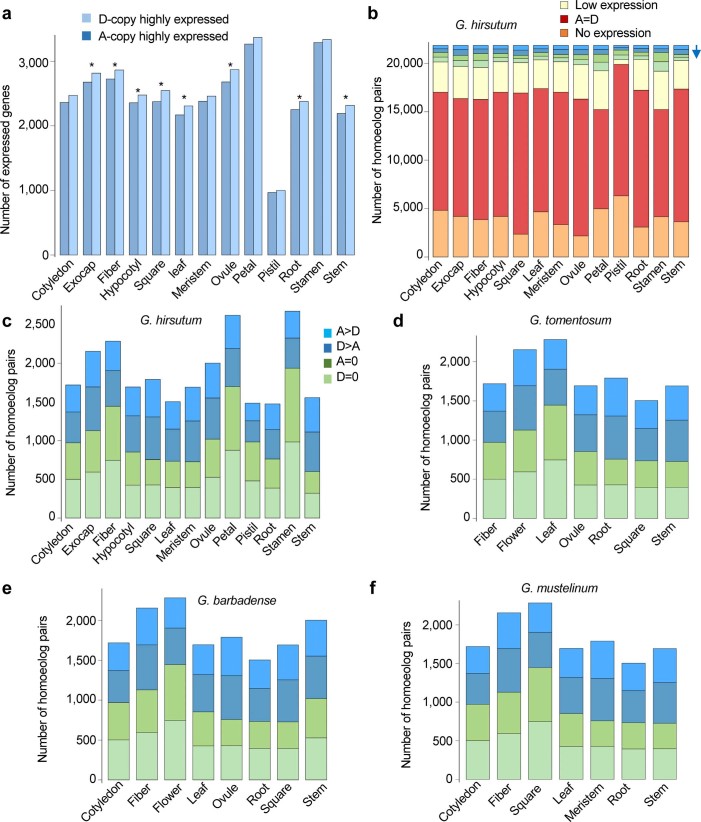
Homeolog expression differences in four allotetraploid cotton species. **a**, Expression levels of homoeologs were compared among different tissues in each speces. The number of homoeologous genes that are more highly expressed (log_2_-fold change ≥1, Benjamini-Hochberg adjusted *P* < 0.05; Wald test) in the A or D subgenome. Asterisks indicate *P* < 0.05 (two-sided binomial test). **b**, Classification of homoeologous pairs by expression patterns. The downward arrow marks the fraction that shows differential expression in different tissues of four species. **c**-**f**, Number of homoeolog pairs (*y* axis) whose expression levels are A > D (pale blue), D > A (dark blue), sub- or neo-funcationalization in A (dark green) or in D (pale green) in *G. hirsutum* (**c**), *G. tomentosum* (**d**), *G. barbadense* (**e**), and *G. mustelinum* (**f**). Tissue types are shown in *x* axis. *G. darwinii* was not included in the analysis due to a small number of tissue types available for the study. Source data

In the five allotetraploid species sequenced, gene expression diversity is dynamic and pervasive across developmental stages and between subgenomes (Supplementary Dataset 9). Principal component analysis shows clear separation of expression between developmental stages (PC1) and between subgenomes (PC3; Extended Data Fig. 6a), with more D homoeologs expressed than A homoeologs in most tissues examined (Extended Data Fig. 7), consistent with higher levels of tri-methylation of Lys 4 on histone H3 (H3K4me3) in the former than in the latter^41^. Notably, expression correlates more closely with the subgenomic variation than with tissue types, except for fiber elongation and cellulose biosynthesis, where subgenomic expression patterns are more closely correlated between Upland and Pima cottons (Extended Data Fig. 6b). This may suggest that domestication drives parallel expression similarities of fiber-related genes in the two cultivated species.

**Extended Data Fig. 8:**
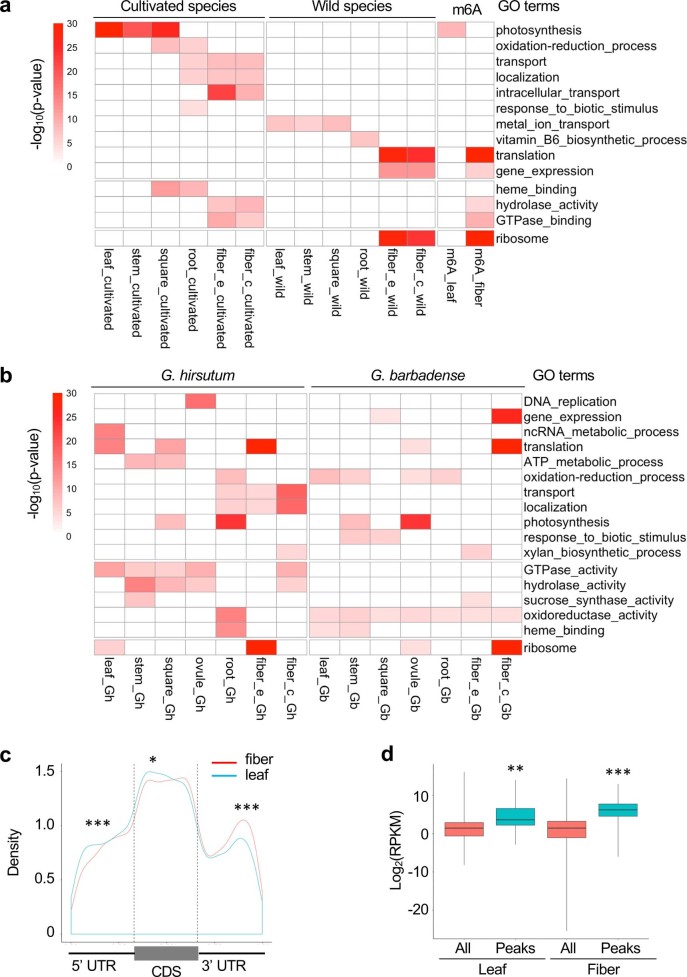
Gene Ontology (GO) analysis of differentially expressed genes and analysis of m^6^A mRNA modifications in Upland cotton. **a**, GO analysis of upregulated genes in two cultivated cottons and three wild relatives (>2-fold change, FPKM > 5, and ANOVA p-value < 0.05) and m^6^A-associated genes in the leaf and fiber of Upland cotton. Color bars = -log_10_(p-value). **b**, GO analysis of upregulated genes (>2-fold change, FPKM > 5, and ANOVA p-value < 0.05) in different tissues of *G. hirsutum* and *G. barbadense*. Color bars = -log_10_(p-value). **c**, Density of m^6^A marks in the genic region, 5ʹ and 3ʹ UTR of ethe xpressed genes in the fiber (red) and leaf (green). Student’s *t*-test was used to compare between m^6^A immuno-precipitated and fragmented (control) RNA reads with single (*) and triple (***) asterisks indicating statistical significance levels of P < 0.05 and <0.001, respectively. **d**, Expression levels (*y* axis) of the genes with m^6^A peaks in the leaf (161 genes) and fiber (1,205 genes) (green), relative to all homoeologous genes (red). Student’s *t*-test was used to compare between m^6^A-associated genes and all homoeologous genes with double (**) and triple (***) asterisks indicating statistical significance levels of P < 0.01 and <0.001, respectively. Source data

These differentially expressed genes in fibers may contribute to fiber development, as they show enrichment of GO groups in hydrolase and GTPase-binding activities (Extended Data Fig. 8a,b). Hydrolases are essential for plant cell wall development^42^, and Ras and Ran GTPases are implicated in the transition from primary to secondary wall synthesis in fibers^43^. Moreover, translation and ribosome biosynthesis pathway genes are enriched during fiber elongation in Upland cotton and during cellulose biosynthesis in Pima cotton, consistent with faster fiber development in Upland cotton and longer fiber duration in Pima cotton^44^.

### Expression networks and m^6^A RNA in fibers

Gene expression diversity is also reflected by coexpression modules in fibers among four species (Supplementary Dataset 10 and Supplementary Note). These module-related genes show higher semantic similarities between domesticated cottons (Gh–Gb) than with two wild species (Gt and Gm). The modules include supramolecular fiber organization genes in Upland cotton and brassinosteroid signaling genes in Pima cotton, which could affect fiber cell elongation^45^. The two wild species have different biological functions and transcription factors enriched in fiber-related gene modules (Supplementary Dataset 11), which may account for the fiber traits that are very different from those of the domesticated species (Fig. 1a).

Transcriptional and post-transcriptional regulation, including the activity of small RNAs and DNA methylation, mediates fiber cell development^46^. Modification of m^6^A messenger RNA can stabilize mRNA and promote translation with a role in developmental regulation of plants and animals^47^. In Upland cotton, m^6^A peaks are found largely in the 5ʹ and 3ʹ untranscribed regions (Extended Data Fig. 8c) of 1,205 genes in developing fibers (Supplementary Dataset 12), at levels 7-fold more than in leaves (Extended Data Fig. 8d) (*P* < 0.002, Student’s *t*-test), while the number of expressed genes is similar in both tissues. Notably, both m^6^A-modified mRNAs and transcriptome data in the fibers target the genes involved in translation, hydrolase activity and GTPase-binding activities (Extended Data Fig. 8a). These results indicate that mRNA stability and translational activities may determine fiber elongation and cellulose biosynthesis when cell cycles arrest in fiber cells.

### Recombination and epigenetic landscapes

**Fig. 4:**
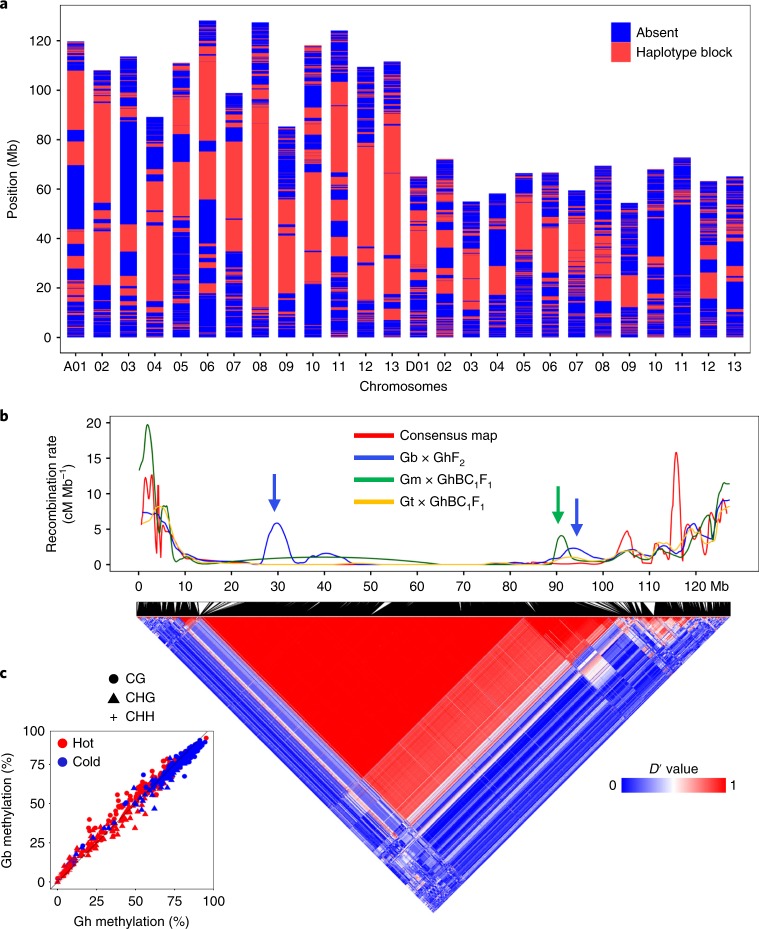
Low-recombination haplotype blocks and their stability and selection during breeding and domestication. **a**, Distribution of presence (red) or absence (blue) of low-recombination haplotype blocks (red) in each pseudo-molecule of the A (A01–A13) and D (D01–D13) subgenomes in Gh. Map positions (Mb) are indicated in the *y* axis. **b**, A low-recombination haplotype block near the pericentromeric region (~72 Mb) of chromosome A08 (bottom). The color indicates the coefficient of linkage disequilibrium (*D*′) from low (blue) to high (red) with the upper confidence bound (*D*′ = 0.90) for the recombination cutoff. The recombination rates (*y* axis; using locally estimated scatterplot smoothing (LOESS) regression, Methods and Supplementary Note) in Gb × GhF_2_ (blue), Gm × GhBC_1_F_1_ (green), Gt × GhBC_1_F_1_ (yellow) and the consensus (red) are shown above with the positions (Mb, *x* axis). Two elevated recombination events are detected in Gb × GhF_2_ (blue arrows) and one in Gm × GhBC_1_F_1_ (green arrow). **c**, The average percentage (%) of CG (circle), CHG (triangle) and CHH (cross) methylation in the recombination hotspots (red) and cold spots (blue) between Gb and Gh. The CHH methylation is clustered in the left lower corner, which is visible in an enlarged image (Extended Data Fig. 10a). Source data

**Extended Data Fig. 9:**
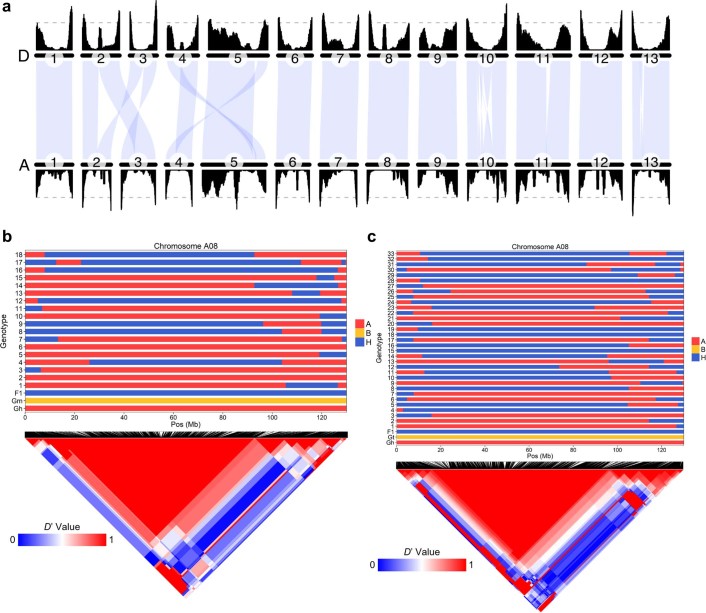
Recombination rate distribution in *G. hirsutum* and inheritance of haplotype blocks in two breeding populations. **a**, Recombination rate distribution between A and D subgenomes. The recombination bins are based on overlapping 5-Mb windows. The dashed grey lines indicate 50% of individuals recombined in the window. The pale blue polygons link syntenic regions. The *x* axis is scaled independently for each homoeologous chromosome. **b**, Linkage disequilibrium heatmap of chromosome A08 of the *G. hirsutum*X*G. mustelinum* BC_2_F_1_ population. Genotypes of 18 lines each representative of one family, two parents, and F_1_ are shown using the CottonSNP63K array (top panel). Red, yellow, and blue colors show the genotypes homozygous for *G. hirsutum*, homozygous for *G. mustelinum*, and heterozygous for both species, respectively. Heatmap (bottom panel) consists of equidistant tiles that indicate linkage disequilibrium as determined by a normalized coefficient of linkage disequilibrium (*D*’) between pairs of markers. Markers corresponding to SNP positions above the heatmap are congruent to the introgressed genotypes (*x* axis). **c**, Linkage disequilibrium heatmap of chromosome A08 of the *G. hirsutum*X*G. tomentosum* BC_3_F_1_ population. Genotypes of 33 lines each representative of one family, two parents, and F_1_ are shown using the CottonSNP63K array (top panel). Red, yellow, and blue colors show the genotypes homozygous for *G. hirsutum*, homozygous for *G. tomentosum*, and heterozygous for both species, respectively. Heatmap (bottom panel) consists of equidistant tiles that indicate linkage disequilibrium as determined by a normalized coefficient of linkage disequilibrium (*D*’) between pairs of markers. Markers corresponding to SNP positions above the heatmap are congruent to the introgressed genotypes (*x* axis). Source data

Polyploidy leads to low genetic recombination, as observed in *B. napus*^48^, which may comprise bottlenecks for breeding improvement. To determine the recombination landscapes in polyploid cottons, we genotyped 17,134 SNPs using the new Gh sequence and the CottonSNP63K array^49^ and identified a total of 1,739 low-recombination haplotype blocks (cold spots) in Upland cotton using whole-genome population-based linkage analysis^50^ (Methods and Supplementary Note). These blocks (average ~678.9 kb with 8.4 SNPs) span 1.18 Gbp (~52%) of the genome, including ~58% and ~41% in the A and D subgenomes, respectively (Fig. 4a), and are dispersed among all chromosomes with large ones predominately near pericentromeric regions. Recombination is generally suppressed throughout haplotype blocks, in contrast to that in subtelomeric regions (Extended Data Fig. 9a).

Chromosome A08 has 62 haplotype blocks, including an exceptionally large one (~72 Mb) (Fig. 4b). Interestingly, interspecific hybridization between different tetraploids can increase recombination rates in these regions. For example, in the Gb × GhF_2_ population, recombination rates increased more than 4–6 cM Mb^−1^ in the left region (29–30 Mb) and in two other regions in the same Gb × GhF_2_ population. Recombination rates were also increased in the Gm × GhBC_1_F_1_ population (Fig. 4b). Similar increases were observed in the homoeologous D08 low-recombination haplotype blocks in the Gb × GhF_2_ population. Moreover, these haplotype blocks of either parent segregated with expected ratios within the population of Gh × GmBC_2_F_1_ (Extended Data Fig. 9b) or Gh × GtBC_3_F_1_ (Extended Data Fig. 9c). These data suggest the stability and selection of these haplotype regions during domestication and breeding.

**Extended Data Fig. 10:**
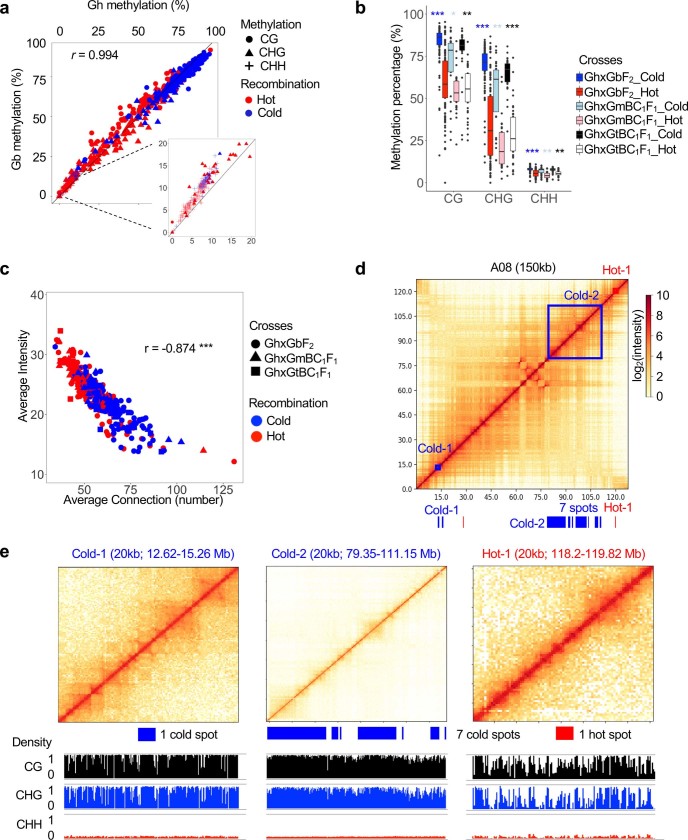
Correlation of DNA methylation levels and chromatin connecting sites and intensities with recombination cold (haplotype block) and hot (no block) spots. **a**, Average percentage (%) of CG (circle), CHG (triangle), and CHH (cross) methylation in the recombination hot (red) and cold (blue) spots between Gb (*y* axis) and Gh (*x* axis), with an enlarged image showing CHH methylation levels. Pearson correlation coefficient is 0.994. **b**, Average methylation percentage (*y* axis) of the recombination spots in different cross in CG, CHG, and CHH sites (*x* axis). Colors indicate recombination hot and cold spots in the three interspecific crosses GhXGbF_2_ (red and blue), GmXGhBC_1_F_1_ (pink and light blue), and GtXGhBC_1_F_1_ (white and black), respectively. ANOVA was used for statistical tests with ingle (*), double (**), and triple (***) asterisks indicating statistical significance levels of P-value<0.001, <1e-5, and <1e-10, respectively. **c**, Chromatin interaction matrices show correlation of chromatin connecting intensity (*y* axis, cutoff >5) with average chromatin connecting numbers (*x* axis, 20-Kb window) of recombination hot (red) and cold (blue) spots in the three interspecific crosses, GhXGbF_2_ (circles), GmXGhBC_1_F_1_ (triangles), GtXGhBC_1_F_1_ (squares). Pearson correlation coefficient is -0.874 with triple (***) asterisks indicating the statistical significance level of P-value<1e-10 (Student’s *t*-test). **d**, Comparison of Hi-C interaction matrix (log2-intensity) in chromosome A08 of the GbXGhF_2_ cross, consisting of recombination hot (red) and cold spots (blue). Locations for one hot spot and two cold spots are shown. **e**, Zoom-in images of two cold and one hot spots in Hi-C interaction matrix (log2 intensity) in chromosome A08, consisting of recombination hot (red) and cold spots (blue), with CG (black), CHG (blue), and CHH (red) methylation densities (100-kb sliding windows). Values at the top of the heatmap represent Hi-C window size (20-kb) and genomic locations (Mb). Gh: *G. hirsutum*; Gb: *G. barbadense*; Gt: *G. tomentosum*; Gm: *G. mustelinum*. Source data

Notably, genome-wide recombination cold spots (haplotype block) and hotspots (no haplotype block) correlated with the DNA methylation frequency at CG, CHG (H = A, T or C) and CHH sites in the cultivated allotetraploids Gh and Gb (Pearson *r* = 0.994; Fig. 4c and Extended Data Fig. 10a,b), with higher methylation frequencies in the cold spots than in the hotspots (analysis of variance (ANOVA), *P* < 1-10e). The data support the role of DNA methylation in altering recombination landscapes, as reported in *Arabidopsis*^51,52^. Consistent with this notion, DNA methylation changes that are induced in the interspecific hybrid (Ga × Gr) are also largely maintained in the five allotetraploid cotton species, creating hundreds and possibly thousands of epialleles, including the ones responsible for photoperiodic flowering and worldwide cultivation of cotton^53^.

Moreover, recombination events in all three interspecific crosses (Gb × GhF_2_, Gm × GhBC_1_F_1_ and Gt × GhBC_1_F_1_) correlated negatively with the average numbers of strongly connecting sites (intensity > 5) (*P* < 8.842 × 10^−16^) and their connection intensities (*P* < 7.26 × 10^−12^) of the Hi-C chromatin matrix (Pearson *r* = −0.874; Extended Data Fig. 10c). Recombination hotspots have fewer but more intense chromatin interactions within short distances, while the cold spots tend to have more but weaker interactions in long distances (Extended Data Fig. 10c,d). For example, 2 hotspots and 9 cold spots in the A08 region (Extended Data Fig. 10d), including 7 cold spots spanning ~32 Mb correlated with weak Hi-C intensities and DNA hypermethylation (Extended Data Fig. 10e). These data indicate that DNA hypermethylation and weak chromatin interactions interfere with recombination events in polyploid cottons.

## Discussion

Despite wide geographic distribution and diversification, five allotetraploid cotton genomes have largely retained the gene content and genomic synteny relative to respective extant diploids. This level of genome stability is in contrast to rapid genomic changes observed in some newly formed allotetraploids such as *B. napus*^10^ and *T. miscellus*^11^. However, in cultivated canola, the two subgenomes are relatively undisrupted^8^, probably because the extant parental species existing today to make new tetraploids^10^ may be different from the ones that formed cultivated canola ~7,500 years ago^16^ and likely became extinct. In addition, all five cotton polyploid species have a monophyletic origin, which is similar to the origin of wild and domesticated tetraploid peanuts^54^, but different from recurrent formation of *Tragopogon* tetraploids^55^. Notably, since polyploid formation 1–1.5 Ma, the evolution of 2 subgenomes in each of the 5 allotetraploid cotton species does not exhibit a simple asymmetrical pattern, as reported in Upland cotton^25^. Instead, the two subgenomes have diversified and experienced novel heterogeneous evolutionary trajectories, including partial equilibration of subgenome size mediated by differential TE exchanges, pervasive evolutionary rate shifts, and positive selection between homoeologs within and among lineages. These features present in all five allotetraploid species suggest that the ‘evolutionary tape’ is replayed during polyploid diversification and speciation.

Among the five allotetraploid genomes, no species-specific orthologs were identified, except for one set of the unique genes related to fiber and seed traits in the two domesticated cottons and another set of the unique genes for reproduction and adaptation in the three wild polyploid species. However, R-gene families have rapidly evolved in each allotetraploid and extensively diversified during selection and domestication. These genomic diversifications have been accompanied by dynamic and prevalent gene expression changes during growth and development between wild and cultivated polyploid species, including parallel gene expression, coexpression networks and m^6^A mRNA modifications in fibers of the cultivated species. Remarkably, polyploid cotton genomes show recombination suppression or haplotype blocks, which correlate with altered epigenetic landscapes and can be overcome by wild introgression and possibly epigenetic manipulation. This finding is contemporary to the discovery of the *Ph1* locus that inhibits pairing of homoeologous chromosomes in polyploid wheat^56,57^. The recombination suppression may help maintain a repository of epigenes or epialleles that were generated by interspecific hybridization accompanied by polyploidization and could have shaped polyploid cotton evolution, selection and domestication^53^. These conceptual advances and genomic and epigenetic resources will help improve cotton fiber yield and quality as a sustainable alternative to petroleum-based synthetic fibers. Modifying epigenetic landscapes and using gene-editing tools may also overcome the limited genetic diversity within polyploid cottons. These principles may facilitate future efforts to concomitantly enhance the economic yield and sustainability of this global crop and possibly other polyploid crops.

## Methods

### Plant materials

*G. hirsutum* L. acc. TM-1 (1008001.06), *G. barbadense* L. acc. 3-79 (1400233.01), G. *tomentosum* L. (7179.01,02,03), *G. darwinii* L. (AD5-32, no. 1808015.09) and *G. mustelinum* L. (1408120.09, 1408120.10, 1408121.01, 1408121.02, 1408121.03) were grown in a greenhouse in College Station at Texas A&M University. Young leaves were collected for preparation of high-molecular-weight DNA using a published method^58^. Total RNA was extracted from leaf, root, stem, square, cotyledon, hypocotyl, meristem, petal, stamen, exocarp, ovule (0, 3, 7, 14, 21 and 35 days post anthesis (DPA)) and fiber (7, 14, 21 and 35 DPA) tissues in Gh; from leaf, root, stem, square, cotyledon, flower, ovule (14 DPA) and fiber (14 DPA) tissues in Gb; from leaf, root, stem, square, cotyledon and fiber (14 DPA) tissues in Gm; from leaf, root, stem, square, flower, ovule (0, 7, 14, 21 and 28 DPA) and fiber (7, 14, 21 and 28 DPA) tissues in Gt; and from leaf, root and stem tissues in Gd. Two or three biological replicates were used for RNA-seq and m^6^A RNA-seq analyses.

### Genome sequencing and assembly

Sequencing reads were collected using Illumina HiSeq and NovaSeq and PacBio SEQUEL and RSII platforms. We sequenced and assembled five *Gossypium* genomes using high-coverage (>74×) single-molecule real-time long-read sequencing (Pac Biosciences). A total of six Illumina libraries were sequenced using the HiSeq platform, and two libraries were sequenced using NovaSeq. Initially, all five species were assembled using MECAT^59^ and subsequently polished using long reads, as well as Illumina reads. Gb and Gh were polished using QUIVER^60^, while Gd, Gt and Gm were polished using ARROW^60^. Ten Hi-C libraries were sequenced for five cotton genomes (two for each species). The total amount of Illumina sequenced for all 5 species (Supplementary Dataset 1) is 4,361,212,302 reads for a total of 286.4× of high-quality Illumina bases. A total of 105,182,984 PacBio reads were sequenced for all 5 genomes with a total coverage of 439.61×.

Chromosome integration of Gb and Gh leveraged a combination of published Gh synteny and Hi-C scaffolding. A total of 148,239 unique, non-repetitive, non-overlapping 1-kb sequences were extracted from the published Gh genome^25^ and aligned to the Gh and Gb MECAT assemblies. Misjoins in the MECAT assembly were identified, and the assembly was scaffolded with Hi-C data using the JUICER pipeline^61^. Small rearrangements to both genomes were made using the JUICEBOX interface^62^. Finally, a set of 5,275 clones (474.3 Mb total sequence) were used to patch remaining gaps in the Gh assembly. A total of 626 gaps were patched resulting in 1,871,050 base pairs (bp) being added to the assembly. Gd and Gm were integrated into chromosomes using Gb (3-79) synteny, whereas Gt was integrated using the Gh release assembly version 1 https://phytozome.jgi.doe.gov/pz/portal.html#!info?alias=Org_Ghirsutum_er. Final refinements to the Gt assembly were made using the JUICER/JUICEBOX pipeline^61^. In all five of the assemblies, care was taken to ensure that the telomere was properly oriented in the chromosomes, and the resulting sequence was screened for retained vector and/or contaminants. Genome annotation and gene prediction procedures are provided in the Supplementary Note.

Dot plots (pairwise comparisons) were generated using Gepard version 1.30 (ref. ^63^). The input data consist of 2 FASTA files, as well as the appropriate flags (-seq1 FASTA_FILE_1 -seq2 FASTA_FILE_2 -matrix edna.mat -zoom 65000 -word 18 -lower 0 -upper 20 -greyscale 0 -format png), with the -zoom flag from 65,000 (D subgenome) to 119,000 (A subgenome). The edna.mat file is part of the Gepard version 1.30 release. As a rule of thumb, this factor is generated by dividing the number of bases of the input FASTA file by 1,000. The output from the Gepard command is a PNG image file.

Procedures for the analysis of SNPs and indels are provided in the Supplementary Note.

### Comparative analysis with published assemblies

#### Assessment of genome completeness

We evaluated the genome assembly completeness by k-mer masking (24-nucleotide) reciprocally between Gh (TM-1)^22^ and Gh (TM-1, this study) and between Gb (Hai7124)^22^ and Gb (3-79, this study). The unmasked contiguous sequences of the unshared sequence were extracted into a FASTA file and analyzed using FASTA statistics. BBMap (https://sourceforge.net/projects/bbmap) and Custom Python scripts (Supplementary Note) were used for this analysis.

#### Genome comparisons using Hi-C data

The Hi-C libraries IKCF (Gh) and ILDE (Gb) were aligned to published Gh and Gb reference genomes using BWA-MEM^64^. Heatmaps were generated using the JUICER-pre command, and visualized using JUICEBOX^62^. Inversions and rearrangements were further identified using JUICEBOX.

#### Analysis of chromosomal collinearity, structural rearrangements and gene family composition between reference assemblies

Published Gh and Gb assemblies^22^ were aligned to the assemblies generated in this study using Minimap2 (ref. ^65^) with the parameter setting ‘-ax asm5 --eqx’. The resulting alignments were used to identify structural rearrangements and local variations using SyRI^66^. The gene copy numbers and gene families between assemblies were identified using OrthoFinder^67^ based on all annotated protein-coding sequences.

### Analysis of evolutionary rate changes and gene gain and loss

#### Evolutionary rate changes in subgenomes of allopolyploid cotton during diversification

Rates of evolution for each subgenome of each species across the phylogeny were calculated using pairwise p-distances for the same 17,136 orthologs in all 5 polyploid species (Extended Data Fig. 4a). The distribution of p-distances between each species was compared for both subgenomes using a one-tailed Wilcoxon signed rank test and Bonferroni correction for multiple testing. Differences in evolutionary rates between the subgenomes within each species were evaluated using a modified relative rate test whereby p-distance distributions were compared for both subgenomes to determine which had the greater p-distance (that is, higher inferred rate). Differences in subgenome evolutionary rates among lineages were estimated using a modified relative rate test that again used the Wilcoxon signed rank test with the p-distances of 17,136 genes, here comparing p-distances between two species relative to an outgroup species. This test was repeated for all possible pairs of tip and outgroup combinations. We also summed the total number of differences contained within all orthologs between each pairwise set of species, excluding all sites in which any of the orthologs contained a gap sequence (Supplementary Dataset 2a). Chi-square tests were used to determine the significance of these total substitution counts (Supplementary Dataset 2b).

#### Analysis of gene loss and gain after polyploid cotton formation

A total of 32,622 groups of SCOs were identified between subgenomes of all 5 allopolyploids and the diploids Gr and Ga (Extended Data Fig. 4c). Of those, the 4,369 SCO groups that were present in both diploid species but absent in at least 1 allopolyploid subgenome were evaluated for gene losses specific to allopolyploids. The list of SCO groups was converted into a binary matrix of gene occurrence and mapped onto the inferred phylogeny of ten allopolyploid subgenomes (with five taxa each in the At- and Dt-subgenome clades, rooted by the respective diploid progenitors). Using a likelihood‐based mixture model assuming predominantly gene losses over gains and stochastic mapping implemented in GLOOME^68^, both the total number of gene gains and losses per branch and the associated probability of each event across the phylogeny were estimated.

#### Identification of homoeologs under selection

The homoeolog pairs of five species were used for estimating non-synonymous/synonymous (*K*_a_/*K*_s_) values. Every pair of the sequences were aligned using the MUSCLE alignment software^69^ and then transferred to the AXT format for identifying positively selected genes (*K*_a_/*K*_s_ > 1) using the KaKs calculator^70^. Positively selected genes in A and D homoeologs were compared pairwise among 5 species (Supplementary Dataset 2).

### Analyses of repetitive sequences and TEs

Pairwise comparison of 18-nucleotide sequences between homoeologous chromosomes was performed by Gepard plots^63^. Analysis of the *k*-mer content of all of the genomes was conducted by LTR-harvest^71^ according to the manual. The whole-genome sequences were suffixed first and then indexed using the seed length 20. The frequency of individual 20-nucleotide sequences was estimated using in-house Perl scripts. This analysis was applied to the two diploid cotton species, Ga and Gr, and the five tetraploid allopolyploids, with the A or D subgenome examined separately. The software LTR-harvest^71^ and LTR-finder^72^ was used for identifying full-length LTR retrotransposons. The identification parameters were as follows. For LTR-harvest: overlaps best -seed 20 -minlenltr 100 -maxlenltr 2000 -mindistltr 3000 -maxdistltr 25000 -similar 85 -mintsd 4 -maxtsd 20 -motif tgca -motifmis 1 -vic 60 -xdrop 5 -mat 2 -mis -2 -ins -3 -del -3. For LTR-finder: -D 15000 -d 1000 -L 7000 -l 100 -p 20 -C -M 0.9. The two datasets were integrated to remove false positives using the LTR-retriever packages^73^. The insertion time was estimated using the formula *T* = *K*_s_/2*r*, where *K*_s_ is the divergence rate and *r* (3.48 × 10^−9^) is the substitution rate in cotton^17^.

Full-length TE sequences were extracted from each of the seven species and were used to build a TE database; the cd-hit software^74^ was applied to remove redundancies through self-sequence similarity tests, and sequences with identity > 90% were grouped into the same cluster. A cluster present in only one species was defined as a species-specific TE cluster, and those present in more than one species were considered shared TE clusters. A total of 98,794 full-length LTRs were identified in all 7 cotton species and grouped into 20,583 clusters for analysis of their origins in Ga, Gr, and the A and D subgenomes in 5 allotetraploids.

### R-gene family and expression analysis in response to pathogen treatments

We detected nucleotide-binding site, leucine-rich repeat (NBS–LRR) motifs with the pfamscan tool^75^ that uses the hidden Markov model search tool (HMMER) version 3.2.1 (ref. ^76^) by searching primary protein-coding transcripts of each of the 5 allotetraploid cottons against the raw hidden Markov model for the NB-ARC-domain family downloaded from Pfam (PF00931). Identified NBS–LRR protein-coding genes for each of the allotetraploid cottons were further analyzed for amino-terminal (TIR/coiled-coil/other) and other functional domains by searching them against the Pfam-A hidden Markov model with the PfamScan tool and HMMER version 3.1 (ref. ^76^) with default settings (Supplementary Note). Short-read sequencing data for bacterial blight were downloaded from the Sequence Read Archive from the NCBI Bioproject accession PRJNA395458 (ref. ^40^). Reniform nematode sequence data were downloaded from the NCBI Bioproject accession PRJNA269348. Sequence data were aligned to the 653 predicted R genes from the Gh version 2.0 (this study) with Bowtie2 version 2.3.4.1 and filtered for true-pair alignments. Fragments per kilobase million (FPKM) and read counts per million were determined with RSEM version 1.3.0. Differentially expressed R genes were determined with edgeR^77^ using false discovery rate (FDR)-corrected *P* values of 0.05. Of the 291 A-subgenome and 384 D-subgenome predicted R genes, we found FPKM expression profiles (>1) for at least 1 condition in 281 and 372 of the A- and D-subgenome predicted R genes, respectively. Similarly, in response to reniform nematode challenge in Gh, 274 of 291 A-subgenome and 370 of 384 D-subgenome predicted R genes were expressed at the FPKM level (>1) for at least 1 of the 4 conditions tested.

### RNA-seq library construction, sequencing and data normalization

Total RNA was extracted from leaf, root, stem, square, flower, ovule and fiber samples from Gh, Gb, Gt, Gm and Gd species (2 replicates each for 124 samples; Supplementary Dataset 9), using PureLink Plant RNA Reagent (ThermoFisher). After DNase treatment, RNA-seq libraries were constructed using an NEBNext Ultra II RNA Library Kit (NEB), and 150-bp paired-end sequences were generated using an Illumina Hiseq 2500.

Paired-end sequence data were quality trimmed (*Q* ≥ 25) and reads shorter than 50 bp after trimming were discarded. Sequences were then aligned to respective allotetraploid cotton genomes and counts of reads uniquely mapping to annotated genes were obtained using STAR (version 2.5.3a). Outliers among the biological replicates were verified on the basis of the Pearson correlation coefficient, *r*^2^ ≥ 0.85. Fragments per kilobase of exon per million (FPKM) fragments mapped values were calculated for each gene by normalizing the read count data to both the length of the gene and the total number of mapped reads in the sample and considered as the metric for estimating gene expression levels^78^. Normalized count data were obtained using the relative logarithm expression (RLE) method in DESeq2 (version 1.14.1)^79^. Genes with low expression were filtered out, by requiring ≥2 RLE-normalized counts in at least 2 samples for each gene. Additional data for RNA-seq expression in fiber (28 DAP) tissue in both Gh and Gb were downloaded from the published data^44^ and processed as described above and in the Supplementary Note.

### Statistical analysis of differentially expressed genes

To measure the gene expression differences between homoeologous genes in RNA-seq data, we used the DESeq2 package in R based on the negative binomial distribution (Supplementary Note). Only genes with log_2_[fold change] ≥ 1, Benjamini–Hochberg-adjusted *P* < 0.05 were retained. The comparison of highly expressed homoeologous gene pairs between subgenomes in different tissues was carried out using a binomial test (*P* < 0.05). GO enrichment was analyzed using topGO^80^, an R Bioconductor package with Fisher’s exact test; only GO terms with *P* < 0.05 (FDR < 0.05) were considered significant.

### Principal component analysis and correlation coefficient analysis

To visualize subgenome and tissue expression relatedness, we used categorized gene expression values. These expression values were averaged across replicates and log_2_-transformed. Principal component analysis employed singular value decomposition via the prcomp function in R^81^. Categorized gene expression values were used in this analysis. Pearson’s correlation coefficients were determined and hierarchical clustering was carried out using the Euclidian distance and complete linkage method.

### m^6^A RNA-seq data analysis

m^6^A RNA-seq libraries were constructed using a modified protocol as previously described^82^. Briefly, total RNA was extracted from young leaf and fiber tissues at 7 DPA (2 replicates each) from Gh by using PureLink Plant RNA Reagent (ThermoFisher). mRNA was collected from total RNA by the Oligotex mRNA mini kit (QIAGEN), fragmented and pulled down using an m^6^A antibody, followed by library construction using the NEBNext Ultra II RNA Library Kit (NEB) without polyA tail selection. Fragmented mRNA-seq libraries (control; input) and m^6^A RNA-seq libraries (IP) were sequenced using an Illumina Hiseq 2500 and 150-bp reads. Illumina reads were mapped to the Gh genome using Tophat 2.1.1 (ref. ^83^), and the uniquely mapped reads were used to identify m^6^A peaks with the Bioconductor package exomePeak^84^ (Supplementary Dataset 12).

GO terms were extracted from the GeneAnnotation_info.txt file. Identified m^6^A peak genes were analyzed by the Bioconductor package topGO^80^ to identify significantly over-represented GO terms (*P* < 0.0001). The location of RNA (5ʹ UTR, CDS or 3ʹ UTR) for each m^6^A RNA-seq read (both input and IP) was identified using the intersect function of Bedtools^85^. Single, double and triple asterisks indicate statistical significance levels of *P* < 0.05, *P* < 0.01 and *P* < 0.001, respectively (Student’s *t*-test).

We extracted the gene expression data for Gh leaf and fiber at 7 DPA corresponding to m^6^A peak genes. ‘All’ refers to the expression level of all identified homoeologous genes in the leaf and fiber samples, while ‘peak’ corresponds to the expression level of the identified m^6^A peaks for the genes in leaf (161 genes) and fiber (1,205 genes) samples. Single, double and triple asterisks indicate statistical significance levels of *P* < 0.05, *P* < 0.01 and *P* < 0.001, respectively (Student’s *t*-test).

### Fluorescence in situ hybridization of A and D homoeologous chromosomes

Procedures for the preparation of metaphase chromosomes in Gh and fluorescence in situ hybridization were adopted from a published protocol^86^, with a modification that the cotton root tips were pretreated with cycloheximide (25 ppm) for 3 h at room temperature. The 25S rDNA fragment was obtained from *Arabidopsis*^87^ and originally provided by R. Hasterok from Poland. Synthetic oligonucleotides for forward and reverse plant telomeric sequences were PCR-amplified and products were labeled by nick translation to create probe to detect telomeres^88^.

### Genotyping and recombination rate analyses

Genotyping data representing an improved cotton panel of 257 Gh accessions were acquired from a previously published diversity analysis^49^ utilizing the CottonSNP63K array^89^. The genotyping data in 2 segregating populations included 18 lines each representing 1 family of a Gh × GmBC_2_F_1_ population and 33 lines each representing 1 family of a Gh × GtBC_3_F_1_ population. SNPs with a minor allele frequency greater than 5% and that had less than 10% missing data were retained. Genotyping data were further filtered for homeo-SNPs that occur due to intragenomic sequence identity^89^. Array ID sequences were aligned to the Joint Genome Institute Gh version 2.0 sequence assembly using BLASTn^90^ (version 2.7.1+) with a minimum e-value cutoff of 1 × 10^−10^. Homoeologous alignments were corrected for using previously published SNP segregation data^89,91^, as well as interspecific, bi-parental linkage mapping populations from their respective Gh × GmBC_1_F_1_ and Gh × GtBC_1_F_1_ initial mapping populations. Genotyping data were then imputed and phased using Beagle (version 4.1)^92^, and genotypes were converted to ABH format to distinguish genotypic parentage.

It is notable that erroneous SNP calling is a common problem in polyploids and especially in the AD-genome allotetraploid cotton because of homoeologous and paralogous sequences. This issue has been addressed through several methods^89,93,94^. In this study, we used the published method^89^ to avoid erroneous genotype calling and to provide accurate chromosome-specific and homoeologous haplotype structure. Furthermore, we used a historical estimation of recombination^95^, as shown in the haplotype structure using confidence intervals, as well as in two segregating populations, which led to the accurate estimates of recombination rates between parental alleles using linkage disequilibrium analysis^95^. The haplotype block partitioning was conducted with PLINK^50^ (Supplementary Note).

The recombination map for chromosome A08 of Gh was developed using 4 SNP-based genetic maps, including 3 of interspecific crosses between Gb × Gh (F_2_, *n* = 195), Gt × Gh (BC_1_F_1_, *n* = 85) and Gm × Gh (BC_1_F_1_, *n* = 59) and 1 consensus map that was generated using 3 intraspecific populations^91^. All genetic maps were aligned to the Joint Genome Institute Gh version 2.0 sequence assembly using the previously stated methods. Recombination map visualization was estimated using the R package MareyMap^96^ using the nonlinear LOESS method^97^, and the number of surrounding markers used to fit a local polynomial was 7.5% of the total number of markers per chromosome. Final map plotting was conducted using the R package ggplot2 (ref. ^98^). Localized recombination rates for chromosomes A08 and D08 were estimated using a 1-Mb non-overlapping sliding window with a minimum of 4 SNPs per window as a linear regression threshold using MareyMap.

### DNA methylation analysis

Methylome sequencing data were downloaded from a published report^53^. In brief, methylC-seq reads of all allopolyploid cottons were mapped to genome sequences of Gh and Gb, respectively, using Bismark with the parameters (--score_min L,0,-0.2 -X 1000 --no-mixed --no-discordant)^99^. Only the uniquely mapped reads were retained and used for further analysis. Reads mapped to the same site were collapsed into a single consensus molecule to reduce clonal bias. Cytosine counts were combined into 1,000-bp windows using methylKit 1.2.4 (ref. ^100^).

The DNA methylation (CG, CHG and CHH) levels (percentage of methylated cytosines) and average Hi-C seq statistics (number of connections, intensity or interaction matrix, and distance) in each recombination spot were compared using custom Python scripts. The Pearson correlation coefficient (*r*) was estimated using singular value decomposition via the prcomp function in R^81^. Single, double and triple asterisks indicate statistical significance levels of *P* < 0.001, *P* < 1 × 10^−5^ and *P* < 1 × 10^−10^, respectively, using one-way ANOVA.

### Chromatin conformation capture (Hi-C) sequencing analysis

Hi-C seq libraries were constructed using a previously described protocol^101,102^, with modifications. Briefly, young leaves from Gh, Gb, Gt, Gm and Gd (2 replicates each) and fiber samples from Gh were fixed in 1% formaldehyde, and nuclei were extracted. Fixed chromatin was digested with DpnII, filled in using biotin-14-dATP and ligated. The biotin-labeled DNA was extracted and pulled down to construct HiC-seq libraries. Sequencing of Hi-C seq libraries was performed using an Illumina Hiseq 2500 and 150-bp reads. Reads were mapped to respective genomes and analyzed by HiC-Pro^103^. The Hi-C read coverage is 205× for Gh, 45× for Gb, 36× for Gm, 22× for Gd and 17× for Gt. The Hi-C data were largely used to correct orientations and misalignments in the assemblies of contigs and scaffolds. For Gh, Hi-C data were used to generate chromatin connection heatmaps with the HiCPlotter (https://github.com/kcakdemir/HiCPlotter). Single, double and triple asterisks indicate statistical significance levels of *P* < 0.001, *P* < 1 × 10^−5^ and *P* < 1 × 10^−10^, respectively, using one-way ANOVA.

### Reporting Summary

Further information on research design is available in the Nature Genetics Research Reporting Summary linked to this article.

## Online content

Any methods, additional references, Nature Research reporting summaries, source data, extended data, supplementary information, acknowledgements, peer review information; details of author contributions and competing interests; and statements of data and code availability are available at 10.1038/s41588-020-0614-5.

## Supplementary information

Supplementary Information Supplementary Note

Reporting Summary

Supplementary Data Twelve supplementary datasets.

## Data Availability

Sequencing data are accessible under NCBI BioProject numbers (PRJNA515894 for Gh, PRJNA516412 for Gt, PRJNA516411 for Gb, PRJNA516409 for Gd and PRJNA525892 for Gm). All datasets generated and/or analyzed in this study are available in the Article, the Source Data files that accompany Figs. 1–4 and Extended Data Figs. 1–10, Supplementary Datasets 1–12, the Reporting Summary or the Supplementary Note. Additional data such as raw image files that support this study are available from the corresponding authors upon request.

## Source data

Source Data Fig. 1 Sequence statistics, genomic features and syntenic relationships.
Source Data Fig. 2 List of genes specific to domesticated cottons and wild species, respectively.
Source Data Fig. 3 TE compositions among five species.
Source Data Fig. 4 Low-recombination haplotype blocks, and their corresponding methylation data.
Source Data Extended Data Fig. 1 Sequence statistics, genomic features and syntenic relationships.
Source Data Extended Data Fig. 2 Copy number variants (CNVs) and structural variations in TM-1 and 3-79 relative to the published data.
Source Data Extended Data Fig. 3 List of Sequence Read Archive files and SCOs for maximum likelihood and coalescent analyses.
Source Data Extended Data Fig. 4 Single-copy orthologs for phylogenetic analysis and for gene loss and gain tests among five species.
Source Data Extended Data Fig. 5 Statistics of TEs between A and D subgenomes among five species and their respective A and D extant diploids.
Source Data Extended Data Fig. 6 RNA-seq gene expression data among different tissues and species.
Source Data Extended Data Fig. 7 RNA-seq expression data for homoeologs in five species.
Source Data Extended Data Fig. 8 GO analysis of the differentially expressed genes among five species and in the fiber and leaf with m^6^A RNA modifications in upland cotton.
Source Data Extended Data Fig. 9 Genomic locations of low-recombination haplotype blocks.
Source Data Extended Data Fig. 10 Comparative analysis for methylome-seq and Hi-C seq data with recombination hotspot and cold-spot distributions.
