# Supplementary material for: Genomic diversifications of five Gossypium allopolyploid species and their impact on cotton improvement
Source: Nat Genet. 2020 Apr 20;52(5):525–33. doi: 10.1038/s41588-020-0614-5 (PMC7203012; doi:10.1038/s41588-020-0614-5)
Supplement: Supplementary file 1 — Supplementary Note [file 41588_2020_614_MOESM1_ESM.pdf]

nature genetics

SUPPLEMENTARY INFORMATION

https://doi.org/10.1038/s41588-020-0614-5

In the format provided by the authors and unedited.

OPEN

Genomic diversifications of five *Gossypium* allopolyploid species and their impact on cotton improvement

Z. Jeffrey Chen[1,2,14] ✉, Avinash Sreedasyam[3,14], Atsumi Ando[1,14], Qingxin Song[1,2,14], Luis M. De Santiago[4,14], Amanda M. Hulse-Kemp[5], Mingquan Ding[1,6], Wenxue Ye[2], Ryan C. Kirkbride[1], Jerry Jenkins[3], Christopher Plott[3], John Lovell[3], Yu-Ming Lin[4], Robert Vaughn[4], Bo Liu[4], Sheron Simpson[7], Brian E. Scheffler[7], Li Wen[8], Christopher A. Saski[8], Corrinne E. Grover[9], Guanjing Hu[9], Justin L. Conover[9], Joseph W. Carlson[10], Shengqiang Shu[10], Lori B. Boston[3], Melissa Williams[3], Daniel G. Peterson[11], Keith McGee[12], Don C. Jones[13], Jonathan F. Wendel[9], David M. Stelly[4], Jane Grimwood[3] ✉ and Jeremy Schmutz[3,10]

[1]Department of Molecular Biosciences, The University of Texas at Austin, Austin, TX, USA. [2]State Key Laboratory for Crop Genetics and Germplasm Enhancement, Nanjing Agricultural University, Nanjing, China. [3]HudsonAlpha Institute for Biotechnology, Huntsville, AL, USA. [4]Department of Soil and Crop Sciences, Texas A&M University System, College Station, TX, USA. [5]US Department of Agriculture-Agricultural Research Service, Genomics and Bioinformatics Research Unit, Raleigh, NC, USA. [6]College of Agriculture and Food Science, Zhejiang A&F University, Lin'an, China. [7]US Department of Agriculture-Agricultural Research Service, Genomics and Bioinformatics Research Unit, Stoneville, MS, USA. [8]Department of Plant and Environmental Sciences, Clemson University, Clemson, SC, USA. [9]Department of Ecology, Evolution, and Organismal Biology, Iowa State University, Ames, IA, USA. [10]The US Department of Energy Joint Genome Institute, Walnut Creek, CA, USA. [11]Institute for Genomics, Biocomputing and Biotechnology and Department of Plant and Soil Sciences, Mississippi State University, Mississippi State, MS, USA. [12]School of Agriculture and Applied Sciences, Alcorn State University, Lorman, MS, USA. [13]Agriculture and Environmental Research, Cotton Incorporated, Cary, NC, USA. [14]These authors contributed equally: Z. Jeffrey Chen, Avinash Sreedasyam, Atsumi Ando, Qingxin Song, Luis M. De Santiago. ✉e-mail: zjchen@austin.utexas.edu; jgrimwood@hudsonalpha.org

Supplementary Note

Methods

Genome annotation pipelines

PERTRAN[1] and PASA[2] were used to produce transcript assemblies from 2×150 paired-end Illumina RNA-seq reads. Repetitive DNA elements were identified *de novo* with repeatModeler[3]. For mapping and genome comparisons, we soft-masked the genomes using repeatMasker[4], with ancestral repeats from RepBase[5] and the repeat annotations from repeatModeler output. Loci were determined by transcript assembly alignments and/or EXONERATE (https://github.com/nathanweeks/exonerate) alignments of proteins from *Arabidopsis thaliana*, soybean, Kitaake rice, sorghum, foxtail millet, *Brachypodium distachyon*, grape and Swiss-Prot proteomes. Gene models were predicted by homology-based methods, FGENESH+ /_EST[6], GenomeScan[7], and AUGUSTUS[8] via BRAKER[9]. The best-scoring predictions for each locus were selected using EST and protein support and penalized if overlapped with repeats. PASA was employed to add UTRs, splicing corrections, and alternative transcripts. High-confidence transcripts were called for loci with BLASTP Cscores >0.5, EST coverage, and <20% of CDS overlapping with repeats (if >20% overlap with repeats, only loci with Cscores >0.9 and homology coverage >0.70 were retained). Gene models with >30% TE domains (Pfam) and those with short single exon (<300 BP CDS), without protein domain or with weak expression evidence were removed.

Completeness of the euchromatic portion of the assembly was assessed using 37,223 *G. raimondii* primary transcripts from the version 2.0 *G. raimondii* release. The transcripts were aligned to the assembly using BLAT and alignments with >=90% base pair identity and >=85% coverage were retained for further analysis.

The D/A gene ratio is ~1.062. An average of 733 annotated genes were in scaffolds, which is largely inflated by Gb with 2,154 genes; otherwise, the average is 378 amongst the four other allotetraploids. There is an average of 5,181 predicted transcription factors among the PCGs, belonging to 58 families. Gt has the highest number (5,212), with an additional 27 transcription factors compared to Gd (5,185).

RepeatMasker estimated repeat content amounts to comprise ~70% of the assemblies with no gaps. Long terminal repeat (LTR) retrotransposons predominated among the repeats.

Phylogenetic analysis

Single copy orthologs (18,698 SCOs or 37,396 homoeologs) present in all polyploid and diploid genomes were used for phylogenetic analysis (Extended Data Fig. 4). SCOs were only selected for phylogenetics if all orthologs were present on a chromosome. The sequence for each gene was recovered using bedtools[10], aligned with MAFFT[11], and gaps were pruned using the R package microseq v1.2.2 with a maximum of 20% gaps on alignment ends and 90% in the alignment interior. Genes were independently subjected to maximum likelihood (ML) phylogenetic analysis using RAxML[12] with rapid bootstrapping, 10,000 bootstrap replicates, and using the general time reversible model (GTRGAMMA) for tree generation. Gene coalescence was estimated separately for each homoeologous tree (i.e., A or D subgenome trees), as well as the combined tree, using an exact search in Astral[13] and outputting full annotation of quartet measurements. Concordance within the coalescence tree was completed using IQtree[14] using both the original set of RAxML trees and gene alignments to calculate concordance for both

genes and sites. A random subset of the same genes were also used for Bayesian phylogenetic analysis using Exabayes[15] with 4 runs of 4 chains and a minimum of 1 million generation to verify consistency with the maximum likelihood based results.

Monophyly in the polyploid clade was evaluated using 1,650 randomly selected homoeologous gene pairs and orthologous sequences from available resequencing in the A- and D- diploid subgenera (Extended Data Fig. 4). Briefly, sequences were downloaded from the short read archive (SRA) for additional accessions and species in the A- and D- subgenera. Reads were mapped against the *G. raimondii* reference genome[16] using gsnap[17] with a diploid cotton specific index to permit SNP tolerant mapping. Orthologous sequences were reconstructed from the mapped reads using bam2consensus[18] and aligned to the polyploid sequences using MAFFT [11]. Alignments were parsed into A- and D- homoeologs (using the alternate diploid as an outgroup), quality trimmed to remove any positions with more the 50% ambiguity among accessions, and individual trees were reconstructed as above.

The species tree was estimated for fractionation analyses (see below) by concatenating homoeologs and conducting a ML analysis in RAxML with the GTRGAMMA model, rapid bootstrapping, and *a posteriori* bootstopping for bootstrap convergence[12]. Diploid sequences were concatenated and used as a single outgroup for this analysis. An additional species tree was constructed which separated A- and D- homoeologs and used *G. kirkii* as an outgroup. Phylogenetic dating in the polyploid clade was constructed using the *chronos* function of {ape}[19] and the rate of molecular evolution derived in[20]. Code related to phylogenetic analyses is found in (Phylogeneticsmethods.txt).

For gene homology analysis, BLAT search from CDS sequences (identity >= 75%; query coverage >= 75%) was used for pairwise comparisons among CDS from five species.The distributions of fourfold degenerate synonymous third-codon transversion rates (4DTv) between single copy paralogous gene pairs between A and D subgenomes within each tetraploid and diploid progenitors (A, D) showed peaks at 0.03 and 0.2, indicative of a recent whole genome duplication and *Gossypium* specific duplication events, respectively. A small shallow peak at ~0.41 in *Theobroma cacao* (Tc) suggests an ancient duplication event. We also compared the distributions of synonymous substitutions per synonymous site (*K*s) for 21,567 collinear orthologous gene sets between A and D subgenomes of allotetraploids, and progenitor-like genomes. Based on *K*s peaks, the divergence time between A and D genomes was estimated to be 4.7 – 5.2 Mya (peaks at 0.033-0.036) and the allotetraploidization event occurred about 0.7–1.2 Mya (*K*s peaks at 0.0047 and 0.0084). However, using a penalized-likelihood based on the concatenated nuclear tree (including branch lengths) as noted above, the divergence is estimated to be 1-1.6 Mya. All tetraploid subgenome comparisons (between At-At and Dt-Dt of each species) showed *K*s peaks at 0.0024 and 0.0033, suggesting that the first divergence among these species occurred about 0.46 – 0.63 Mya. A phylogenetic analysis using 100 single-copy orthologous genes between A and D progenitor genomes, A_t, and D_t subgenomes of five allotetraploids and Tc (as an outgroup), identified the clear branching pattern of A and D genomes with A and D subgenomes of allotetraploids, respectively (i.e., A vs. At; D vs. Dt). Using a reference divergence time of 5-7 Mya between A (Ga) and D (Gr) species, the estimated divergence time between allotetraploids and the diploid progenitors approximately reflected the *K*s based divergence-time estimations. Divergence time [$T = Ks/(2r)$] was estimated using the synonymous substitution rate (r) of 3.48×10^{-9} synonymous substitutions per synonymous site per year that has been calibrated in cotton[20] and 10,852 single copy orthologs across species. *Ks* values >1 were removed to eliminate saturated synonymous sites.

Quantification of homoeolog evolutionary rate changes following polyploidy: To estimate how many genes have experienced a shift in evolutionary rate following polyploidy, we used 18,698 genes present in single copy in *G. raimondii*, *G. arboreum*, and each subgenome of *G. hirsutum, G. barbadense, G. tomentosum, G. mustelinum,* and *G. darwinii* (Extended Data Fig. 4) Coding sequences were aligned using the codon-aware alignment tool MACSE v2[21], and any gene in which one sequence had an inferred frameshift in the alignment was not included in subsequent analyses. The resulting 17,136 pairs of homoeologs were analyzed using the codeml package of PAML v4.9[22] under various molecular clock hypotheses and using the species tree as the gene tree. All tests using PAML were done through the PAML Biopython library v1.70 using Python v3.6.3. Log likelihood scores and rate estimates were extracted from the output, and all comparisons between models were compared using AIC.

We classified genes into a high-confidence set, where the second-best model had a ΔAIC greater than 2, while genes that had a ΔAIC lower than 2 were put into a lower-confidence set. For genes in the lower-confidence set, if a competing model with ΔAIC lower than 2 had fewer parameters, we chose this simpler model as the best model, but kept it in the low-confidence set. After the best model was determined for each gene, we further parsed each model into groups where the shift in rate was faster or slower than the background rate (for models with only a single added parameter) and for the six possibilities of relative rate changes for the model with two additional parameters (Supplementary Dataset 2).

Single nucleotide polymorphism (SNPs) and insertions and deletions (INDELs)

The consensus sequences were used to identify SNPs and INDELs using next-generation sequencing (Illumina, ~286.4X) by aligning the reads with the BWA-MEM https://arxiv.org/abs/1303.3997 and the GATK's tool.

Reads were aligned using BWA-MEM paired alignment functionality[23]. The resulting sam file was converted to a binary (.bam) format and unpaired alignments were removed. The Genome Analysis Toolkit (GATK)[24] was used to remove duplicate reads arising from PCR artifacts and for detailed local realignment to accurately assign INDELs. An initial set of SNPs/INDELs were then called on the remaining alignments using GATK's UnifiedGenotyper. This set of SNPs/INDELs were filtered by GATK's VariantFiltration function, using the following criteria: (a) mapping quality >51.0 and <61.0, (b) variant quality to depth ratio of less than 2.0, (c) Phred-scale p-value of greater than 60 for Fisher's exact test to detect strand bias, (d) GATK haplotype score of <13.0, (e) coverage depth between eight and one hundred, (f) a variant quality score >=50.0, (g) Z-score >-12.5 from the Wilcoxon rank sum test comparing variant and reference read mapping qualities, (h) Z-score >-8.0 from the Wilcoxon rank sum test for comparing variant and reference read position bias, (i) no more than 3 SNPs within 10bp of each other, and (j) variant could not be within 25 bases of a repeat masked region. An upper bound for the mapping quality was included in addition to the lower bound as it is our experience that mapping qualities greater than 60 tend to have a greater number of suboptimal alternative positions and are ambiguous.

SNPs in 120 F2 individuals from the Gh X Gb cross were also used to correct orientation of contigs and scaffolds (Supplementary Dataset 4).

R gene family analysis

We detected nucleotide binding site leucine-rich repeats (NBS-LRR) motifs with the pfamscan tool[25] that uses the Hidden Markov Model search tool (HMMER) v3.2.1[26] by searching primary protein coding transcripts of each of the 5 allotetraploid cottons against the raw hidden markov model for the NB-ARC-domain family downloaded from Pfam (PF00931). Identified NBS-LRR protein coding genes for each of the allotetraploid cottons were further analyzed for N-terminal (TIR/coiled-coil/other) and other functional domains by searching the against the Pfam-A hidden Markov model with the PfamScan tool and HMMER v. 3.1[26] with default settings. NBS-LRR primary gene models were also batch searched against the NCBI Conserved Domain Database to identify consensus conserved domains. The complete set of NBS-LRR genes for each species were aligned with MUSCLE v3.8.31[27]. The number of NBARC genes determined in each subgenome for each tetraploid species was tested for subgenome level dominance. A generalized linear mixed model was run in SAS Statistical Software version 9.4[28] using both discrete number and percentage of total genes with species as replicates. Each species was tested to determine if the number of different gene numbers (overall NBARC, unique NBARC, number of homeologus pairs of shared NBARC) or number of R-gene domain classes (CC, TIR, RPW8, total AAA, AAA_16, AAA_22, PLN03210, PRK15386 superfamily, and AMN1 superfamily) were significantly different than the other tetraploid species. The corresponding number of each was tested in SAS with a generalized linear mixed model with species as class using count weight and binary distribution to compare species lsmeans. Integrated domains, e.g., encoded domains other than characteristic TIR/CC, were determined by filtering R-genes for the TIR/CC domains with the NBCI Conserved Domain Database[29]. Annotated genes associated with high-quality genome references for 6 rosid perennial tree type species (*Malus domestica, Eucalyptus grandis, Carica papaya, Theobroma cacao, Gossypium arboreum, Gossypium raimondii*), *Arabidopsis thaliana*, and one astrid species as an outgroup (*Coffea canephora*) were obtained. Total genes were used to annotate and classify R genes using MATRIX-R[30] according to subdomain classes. Numbers of subdomain R-gene classes were tested for significance among *Gossypium* tetraploids using each species as a replicate and classes assigned based on Order/Family/Clade of each species, using a Generalized Linear Mixed Model in SAS[28].

Transcriptome analysis among five species and tissues and between A and D subgenomes

Reads were mapped to each species genome using Tophat 2.1.1[31], and uniquely mapped reads were used to analyze gene expression by Cufflinks 2.2.1[32]. Differentially expressed genes (DEGs) were identified by >2-fold change and ANOVA p-value <0.05.

Reads corresponding to homoeologous genes among all five species (43,651 genes) were extracted from each RNA-seq sample, and then split them into subgenome A and D paired genes (16536 pairs). In addition, we categorized all RNA-seq samples (homoeologouss genes with A and D pairs) into four categories (Vegetative, Reproductive, Fiber_Elongation, Fiber_Cellulose_biosynthesis), and calculated the average gene expression value of combined samples to conduct further analysis. The Vegetative category consisted of: leaf, stem, and root; Reproductive: ovule and floral organ; Fiber_Elongation: 7 to 21DPA fiber; Fiber_Cellulose_biosynthesis: 28 to 35DPA fiber.

For testing biased expression, paired-end sequence data were quality trimmed ($Q \geq 25$) and reads shorter than 50 bp after trimming were discarded. Sequences were then aligned to respective allotetraploid cotton genomes and counts of reads uniquely mapping to annotated genes were obtained using STAR (v2.5.3a). Outliers among the biological replicates were

verified based on the Pearson correlation coefficient, $r^2 \geq 0.85$. Fragments per kilobase of exon per million fragments mapped (FPKM) values were calculated for each gene by normalizing the read count data to both the length of the gene and the total number of mapped reads in the sample and considered as the metric for estimating gene expression levels[32].

High-confidence homeologous gene pairs were identified by sequence similarity search using BLASTP of all annotated genes in a subgenome against the other and limiting to the gene pair to the syntenic hits from MCScanX[33] and orthogroups obtained from Orthofinder (default settings)[34]. Gene pairs residing on homeologous chromosomes and previously reported reciprocal translocations were considered as homoeologous gene pairs for subgenome expression analysis. We performed differential expression analysis between homeologous pairs for each tissue using DESeq2 (v1.22.2)[35] with a $\log_2$ expression ratio ≥ 1 and Benjamini-Hochberg adjusted P-values < 0.05 as the statistical cutoff for identifying asymmetrically expressed genes. The comparison of highly expressed homoeologous gene pairs between subgenomes in different tissues was carried out using binomial tests, P <0.05 were considered significant.

topGO[36,37], an R Bioconductor package, was used to determine overrepresented GO categories across biological processes (BP), cellular component (CC) and molecular function (MF) domains among differentially expressed genes. Enrichment of GO terms was tested using Fisher's exact test with P < 0.05 considered as significant. Statistical analyses and visualizations were performed using the R 3.5.1 Statistical Software[38].

Co-expression network construction and module detection

Weighted gene co-expression networks were constructed using the WGCNA R package (v1.66)[39] with expression data normalized using variance stabilizing transformation from the DESeq2 R package (v1.22.2)[35]. The data retained after filtering genes showing low expression levels (minimum read count = 6 and minimum total read count = 10) were used to construct co-expression network modules using the block-wise network construction procedures. Briefly, pairwise Pearson correlations between each gene pair were weighted by raising them to power (β). To select a proper soft-thresholding power, the network topology for a range of powers was evaluated and appropriate power was chosen that ensured an approximate scale-free topology of the resulting network. The pairwise weighted matrix was transformed into topological overlap measure (TOM). And the TOM-based dissimilarity measure (1 – TOM) was used for hierarchical clustering and initial module assignments were determined using a dynamic tree-cutting algorithm. Pearson correlations between each gene and each module eigengene, referred to as a gene's module membership, were calculated and module eigengene distance threshold of 0.25 was used to merge highly similar modules. These co-expression modules were assessed to determine their correlation with expression patterns distinct to tissues. Interesting modules having significant relationships with tissues, such as fiber, were visualized using the igraph R package (v1.2.4)[40] and in order to focus on the relevant gene pair relationships, network depictions were limited to an adjacency threshold of 0.2 and the top 3000 edges/interactions between nodes/gene models.

Recombination and haplotype block analysis

Linkage disequilibrium (LD) analysis was conducted using SNPs that were successfully aligned to the reference genome and whose homolog was previously known based on linkage mapping analyses. Haplotype block partitioning was conducted with PLINK[41] using confidence intervals (CI), which classify pairs of markers into one of three LD categories[42]. Default CI

parameters were used with the upper and lower 95% confidence bounds set to 0.98 and 0.70, respectively, and the upper confidence bound of *D*' was set to .90 as evidence for historical recombination. No maximum block length was set in order to conduct chromosome-wide block partitioning. Haplotype heatmaps were generated using HaploView[43] (version 4.2) with identical CI parameters and LD heatmaps were generated using the R package LDheatmap[44].

For sequence-based recombination rate analysis, all shared 50-mers between parents (Gh TM1 and Gb 3-79) were extracted and culled to the loci where the 51st position was unique to each parent. The resulting 4,917,111 pairs of unique 51-mers were counted in the resequencing reads (Illumina 2 x 250 bp) of the 110 F2 individuals in the progeny. To make high-confidence genotype calls, the genotype matrix was split into 40-marker overlapping 50-marker windows. The proportion of parent 1 – unique kmer hits in that window was tabulated for each library and rounded to 1.0 (likely parent1 | parent1 homozygote), 0.5 (1|2) or 0.0 (2|2). These raw genotype calls were iteratively culled to runs of no less than 100 consecutive identical calls. The resultant runs of identical calls are haplotype blocks. To assess the rate of recombination, we counted the number of haplotype block breakpoints within each 900-Kb overlapping 1-Mb window.

Custom Python scripts for genome completeness assessment

```
      from Bio import SeqIO

import re

#===============================================================
def real_main():

    # Unmasked Sequency Puller
    seqPuller = re.compile( r'[ACTGactg]+' ).finditer

    nBases = 0
    for r in SeqIO.parse("MASKED.FASTA", "fasta"):
        tmpSeq = str( r.seq )
        nBases += sum( [tmpSeq.count(item) for item in 'ACTGactg'] )
        for item in seqPuller(tmpSeq):
            start, end = item.span()
            ##print("%s|%d|%d" % ( r.id, start, end ))
        #####
    #####
    print("Bases remaining after masking %s" % nBases)

#===============================================================
if ( __name__ == '__main__' ):
    real_main()
```

References

1. Shu, S., Goodstein, D. & Rokhsar, D. *PERTRAN*: Genome-guided RNA-seq Read Assembler. (United States, 2013).
2. Haas, B.J. et al. Improving the Arabidopsis genome annotation using maximal transcript alignment assemblies. *Nucleic Acids Res* **31**, 5654-66 (2003).
3. Smit, A. & Hubley, R. RepeatModeler Open-1.0. Repeat Masker. (http://www.repeatmasker.org/RepeatModeler/, 2010).

4. Smit, A., Hubley, R. & Green, P. RepeatMasker Open-4.0.6 2013-2015. (http://www.repeatmasker.org, 2017).
5. Jurka, J. et al. Repbase Update, a database of eukaryotic repetitive elements. *Cytogenet Genome Res* **110**, 462-7 (2005).
6. Salamov, A.A. & Solovyev, V.V. Ab initio gene finding in Drosophila genomic DNA. *Genome Res* **10**, 516-22 (2000).
7. Yeh, R.-F., Lim, L.P. & Burge, C.B. Computational inference of homologous gene structures in the human genome. *Genome Res* **11**, 803-816 (2001).
8. Stanke, M., Steinkamp, R., Waack, S. & Morgenstern, B. AUGUSTUS: a web server for gene finding in eukaryotes. *Nucleic Acids Res* **32**, W309-12 (2004).
9. Hoff, K.J., Lange, S., Lomsadze, A., Borodovsky, M. & Stanke, M. BRAKER1: Unsupervised RNA-Seq-Based Genome Annotation with GeneMark-ET and AUGUSTUS. *Bioinformatics* **32**, 767-9 (2016).
10. Quinlan, A.R. & Hall, I.M. BEDTools: a flexible suite of utilities for comparing genomic features. *Bioinformatics* **26**, 841-2 (2010).
11. Katoh, K. & Standley, D.M. MAFFT multiple sequence alignment software version 7: improvements in performance and usability. *Mol Biol Evol* **30**, 772-80 (2013).
12. Stamatakis, A. RAxML version 8: a tool for phylogenetic analysis and post-analysis of large phylogenies. *Bioinformatics* **30**, 1312-3 (2014).
13. Sayyari, E. & Mirarab, S. Fast Coalescent-Based Computation of Local Branch Support from Quartet Frequencies. *Mol Biol Evol* **33**, 1654-68 (2016).
14. Nguyen, L.T., Schmidt, H.A., von Haeseler, A. & Minh, B.Q. IQ-TREE: a fast and effective stochastic algorithm for estimating maximum-likelihood phylogenies. *Mol Biol Evol* **32**, 268-74 (2015).
15. Aberer, A.J., Kobert, K. & Stamatakis, A. ExaBayes: massively parallel bayesian tree inference for the whole-genome era. *Mol Biol Evol* **31**, 2553-6 (2014).
16. Paterson, A.H. et al. Repeated polyploidization of Gossypium genomes and the evolution of spinnable cotton fibres. *Nature* **492**, 423-7 (2012).
17. Wu, T.D. & Nacu, S. Fast and SNP-tolerant detection of complex variants and splicing in short reads. *Bioinformatics* **26**, 873-81 (2010).
18. Page, J.T., Liechty, Z.S., Huynh, M.D. & Udall, J.A. BamBam: genome sequence analysis tools for biologists. *BMC Res Notes* **7**, 829 (2014).
19. Paradis, E., Claude, J. & Strimmer, K. APE: Analyses of Phylogenetics and Evolution in R language. *Bioinformatics* **20**, 289-90 (2004).
20. Grover, C.E. et al. Comparative genomics of an unusual biogeographic disjunction in the cotton tribe (Gossypieae) yields insights into genome downsizing. *Genome Biol Evol* **9**, 3328-3344 (2017).
21. Ranwez, V., Douzery, E.J.P., Cambon, C., Chantret, N. & Delsuc, F. MACSE v2: Toolkit for the Alignment of Coding Sequences Accounting for Frameshifts and Stop Codons. *Mol Biol Evol* **35**, 2582-2584 (2018).
22. Yang, Z. PAML 4: phylogenetic analysis by maximum likelihood. *Mol Biol Evol* **24**, 1586-91 (2007).
23. Li, H. Aligning sequence reads, clone sequences and assembly contigs with BWA-MEM. *arXiv* **1303**, 3997 (2013).
24. McKenna, A. et al. The Genome Analysis Toolkit: a MapReduce framework for analyzing next-generation DNA sequencing data. *Genome Res* **20**, 1297-303 (2010).

25. Chojnacki, S., Cowley, A., Lee, J., Foix, A. & Lopez, R. Programmatic access to bioinformatics tools from EMBL-EBI update: 2017. *Nucleic Acids Res* **45**, W550-W553 (2017).
26. Eddy, S.R. Accelerated Profile HMM Searches. *PLoS Comput Biol* **7**, e1002195 (2011).
27. Edgar, R.C. MUSCLE: multiple sequence alignment with high accuracy and high throughput. *Nucleic Acids Res* **32**, 1792-7 (2004).
28. SAS Institute Inc. SAS version 9.4. (SAS Institute Inc, Cary, NC, 2013).
29. Marchler-Bauer, A. et al. CDD: NCBI's conserved domain database. *Nucleic Acids Research* **43**, D222-D226 (2015).
30. Sanseverino, W. et al. PRGdb 2.0: towards a community-based database model for the analysis of R-genes in plants. *Nucleic Acids Research* **41**, D1167-D1171 (2013).
31. Kim, D. et al. TopHat2: accurate alignment of transcriptomes in the presence of insertions, deletions and gene fusions. *Genome Biology* **14**(2013).
32. Trapnell, C. et al. Differential gene and transcript expression analysis of RNA-seq experiments with TopHat and Cufflinks. *Nature Protocols* **7**, 562-78 (2012).
33. Wang, Y.P. et al. MCScanX: a toolkit for detection and evolutionary analysis of gene synteny and collinearity. *Nucleic Acids Research* **40**(2012).
34. Emms, D.M. & Kelly, S. OrthoFinder: solving fundamental biases in whole genome comparisons dramatically improves orthogroup inference accuracy. *Genome Biol* **16**, 157 (2015).
35. Love, M.I., Huber, W. & Anders, S. Moderated estimation of fold change and dispersion for RNA-seq data with DESeq2. *Genome Biol* **15**, 550 (2014).
36. Alexa, A., Rahnenfuhrer, J. & Lengauer, T. Improved scoring of functional groups from gene expression data by decorrelating GO graph structure. *Bioinformatics* **22**, 1600-7 (2006).
37. Alexa, A. & Rahenfuhrer, J. topGO: Enrichment Analysis for Gene Ontology. in *R package version 2.32.0* (2016).
38. International Wheat Genome Sequencing, C. et al. Shifting the limits in wheat research and breeding using a fully annotated reference genome. *Science* **361**(2018).
39. Langfelder, P. & Horvath, S. WGCNA: an R package for weighted correlation network analysis. *BMC Bioinformatics* **9**, 559 (2008).
40. Csardi, G. & Nepusz, T. The igraph software package for complex network research. *InterJournal, Complex Systems*, 1695 (2006).
41. Purcell, S. et al. PLINK: a tool set for whole-genome association and population-based linkage analyses. *Am J Hum Genet* **81**, 559-75 (2007).
42. Gabriel, S.B. et al. The structure of haplotype blocks in the human genome. *Science* **296**, 2225-9 (2002).
43. Barrett, J.C., Fry, B., Maller, J. & Daly, M.J. Haploview: analysis and visualization of LD and haplotype maps. *Bioinformatics* **21**, 263-265 (2005).
44. Shin, J., Blay, S., McNeney, B. & Graham, J. LDheatmap: An R Function for Graphical Display of Pairwise Linkage Disequilibria Between Single Nucleotide Polymorphisms. *Journal of Statistical Software* **16**, 1-9 (2006).
