# Supplementary material for: Genomic diversifications of five Gossypium allopolyploid species and their impact on cotton improvement
Source: Nat Genet. 2020 Apr 20;52(5):525–33. doi: 10.1038/s41588-020-0614-5 (PMC7203012; doi:10.1038/s41588-020-0614-5)
Supplement: Supplementary file 2 — Reporting Summary [file 41588_2020_614_MOESM2_ESM.pdf]

# nature research

Corresponding author(s): Z. Jeffrey Chen and Jane Grimwood

Last updated by author(s): Mar 5, 2020

# Reporting Summary

Nature Research wishes to improve the reproducibility of the work that we publish. This form provides structure for consistency and transparency in reporting. For further information on Nature Research policies, see Authors & Referees and the Editorial Policy Checklist.

## Statistics

For all statistical analyses, confirm that the following items are present in the figure legend, table legend, main text, or Methods section.

| n/a | Confirmed | |
|---|---|---|
| ☐ | ☒ | The exact sample size (*n*) for each experimental group/condition, given as a discrete number and unit of measurement |
| ☐ | ☒ | A statement on whether measurements were taken from distinct samples or whether the same sample was measured repeatedly |
| ☐ | ☒ | The statistical test(s) used AND whether they are one- or two-sided<br>*Only common tests should be described solely by name; describe more complex techniques in the Methods section.* |
| ☒ | ☐ | A description of all covariates tested |
| ☐ | ☒ | A description of any assumptions or corrections, such as tests of normality and adjustment for multiple comparisons |
| ☐ | ☒ | A full description of the statistical parameters including central tendency (e.g. means) or other basic estimates (e.g. regression coefficient) AND variation (e.g. standard deviation) or associated estimates of uncertainty (e.g. confidence intervals) |
| ☐ | ☒ | For null hypothesis testing, the test statistic (e.g. *F*, *t*, *r*) with confidence intervals, effect sizes, degrees of freedom and *P* value noted<br>*Give P values as exact values whenever suitable.* |
| ☐ | ☒ | For Bayesian analysis, information on the choice of priors and Markov chain Monte Carlo settings |
| ☒ | ☐ | For hierarchical and complex designs, identification of the appropriate level for tests and full reporting of outcomes |
| ☒ | ☐ | Estimates of effect sizes (e.g. Cohen's *d*, Pearson's *r*), indicating how they were calculated |

*Our web collection on statistics for biologists contains articles on many of the points above.*

## Software and code

Policy information about availability of computer code

**Data collection**

(1) DNA sequencing was performed using Illumina HiSeq 2500, NovaSeq, PacBio - RSII, SEQUEL and corresponding software from the manufacturers.
(2) RNA-seq data and m6A RNA-seq were generated using Illumina - HiSeq 2500 (2X150 bp paired-end reads) and its software.
(3) Methylome (MethylC seq) data were generated using paired-end sequencing for 126 cycles using Illuma HiSeq 2500.
(4) Hi-C sequencing was performed using Illuma HiSeq 2500 (2X150 bp paired-end reads), and reads were mapped using HiC-Pro.
(5) All SNP data were generated by the CottonSNP63K Array, and genotypes were called using GenomeStudio (v2.0).

**Data analysis**

(1) Assembly and annotation: We used MECAT (v1.3), QUIVER (v2.0.0), ARROW (v2.0.0), JUICER (v1.5.6) and JUICEBOX (v1.9.0) for genome assembly. Following tools were used for genome annotation: Augustus (v3.0.3); PERTRAN (v1.0); PASA (v2.3.3); InterProScan (v 5.32-71.0); RepeatModeler (v1.0.11); Repeatmasker (v4.0.5); BUSCO (v2.0); EXONERATE (v2.4.0); FGENESH+; GenomeScan (v1.0); BRAKER (v2.1.2); and BLAT (v35).

(2) Assessment of genome completeness: We evaluated the genome assembly completeness by k-mer masking (24-mer) reciprocally between Gh (Hu et al. 2019) and Gh (this study) and between Gb (Hai 7124, Hu et al. 2019) and Gb (3-79, this study) using BBMap (v38.45). The unmasked contiguous sequences or the unshared sequence were extracted into a FASTA file and analyzed FASTA statistics. Custom Python scripts (Supplementary Dataset 19) were used for this analysis. Genome comparisons using HiC data: HiC libraries IKCF (Gh) and ILDE (Gb) were aligned to published Gh and Gb reference genomes using BWA-MEM. Heatmaps were generated using the JUICER-pre command, and visualized using JUICEBOX. Inversions and rearrangements were further identified using JUICEBOX.

(3) Analysis of chromosomal collinearity, structural rearrangements and gene family composition between reference assemblies: Gh and Gb assemblies (Hu et al., 2019) were aligned to the assemblies generated in this study using Minimap2 with parameter setting “-ax asm5 –eqx”. The resulting alignments were used to identify structural rearrangements and local variations using SyRI. The gene copy numbers and gene families between assemblies were identified using OrthoFinder based on all annotated protein coding sequences.

(4) Analysis of orthologs and homoeologs: We used BLAST+ (2.5.0), diamond (v0.9.21.122) and OrthoFinder (2.0) to identify

homoeologous and orthologous sequences. GO functional enrichment analysis was performed using the topGO R package (2.34.0).

(5) Evolutionary analysis: We used MUSCLE (v3.8.1551), MAFFT (v7.221 and v7.407), RAxML (v8.2.11), ASTRAL (v5.6.3), IQtree (v1.7), MACSE (v2.03), GLOOMe (vMay 2013), and PAML (v4.9i) for phylogenetic analysis and evolutionary rate estimates. The evolutionary time was estimated using the formula T= Ks/2r, where Ks is the divergence rate, and r is the mutation rate in cotton (3.48x10-9). Rates of evolution for each subgenome of each species across the phylogeny were calculated using pairwise p-distances for the same 17,136 orthologs in all five polyploid species. The distribution of p-distances between each species was compared for both subgenomes using a one-tailed Wilcoxon Signed Rank test and Bonferroni correction for multiple testing. Differences in evolutionary rates between the subgenomes within each species were evaluated using a modified relative rate test whereby p-distance distributions were compared for both subgenomes to determine which had the greater p-distance (i.e., higher inferred rate). Differences in subgenome evolutionary rates among lineages were estimated using a modified relative rate test that again used the Wilcoxon Signed Rank test to with the p-distances of 17,136 genes, here comparing p-distances between two species relative to an outgroup species. This test was repeated for all possible pairs of tip and outgroup combinations.

(6) The homeolog pairs of five species were used for estimating non-synonymous/synonymous (Ka/Ks) values. Every pair of the sequences were aligned using the MUSCLE alignment software and then transferred to the AXT format for identifying positively selected genes (PSGs, Ka/Ks>1) using the KaKs Calculator. PSGs in A and D homoeologs were compared pairwise among five species.

(7) R-gene family analysis was determined with the Hidden Markov Model (HMMER v3.2.1) and the PfamScan tool.  MUSCLE v3.8.31 was used for R-gene protein alignments.  R-gene statistical analysis was performed in SAS and classified with MATRIX-R.

(8) RNA-seq analysis of homoeolog expression: We used STAR (v2.5.3a) to map and count the RNA-seq reads against the reference genomes and annotations. DESeq2 (v1.14.1) was used to perform normalization and generate the expression tables and perform differential gene expression analyses. We used bwa (0.7.15-r1140 ) and GATK (4-4.1.2) for variant calling. Samtools (1.9), bedops (v2.4.35), and bedtools (v2.27.1) were used to operate on genomic alignment and coordinate files. For analysis across species and tissues, we used Cufflinks (v2.2.1) for expression analysis and Python (v2.7.15) and NumPy (1.16.1) for calculating ANOVA p-value and average FPKM of replicates. We also used the prcomp and cor function in R (v3.5.1) to conduct principle component analysis (PCA) and Pearsons's correlation coefficiency analysis, respectively. Biocondutor package topGO (v2.36.0) was used for gene ontology analysis.

(9) Co-expression network analysis was performed using WGCNA R package (1.66). Data processing was done using Python 2.7 and Python 3.6, using Biopython library (v1.70). Statistical analyses were done in R (3.5.1) using packages dplyr (0.8.0.1), data.table (1.12.0), microseq (1.2.3) and tidyverse (1.2.1). Plots were created using the R packages ggplot2 (3.1.0), ape (5.3), and ggpubr (0.2).

(10) m6ARNA-seq analysis: We used Tophat (v2.1.1) for mapping, Samtools (v1.5) for extracting uniquely mapped reads, and Biocondutor package exomePeak (v2.17.0) for identifying m6A peaks. We used intersect function of Bedtools (v2.26.0) to identify the location of RNA (5'UTR, CDS, or 3'UTR). Biocondutor package topGO (v2.36.0) was used for gene ontology analysis.

(11) K-mer and TE analyses: The LTR-harvest (function inside the genometools 0.6.5) was used to analyze frequency and distribution of 20-mer repeat sequences in each genome. LTR-finder (v1.07) and LTR-harvest were used to identify full-length retrotransposons. LTR-retriever was used to integrate those TEs generated by both LTR-finder and LTR-harvest, as well as to predict the TE insertion time using the cotton mutation rate (r=3.48x10-9). Violin plots of insertion time were generated using ggplot2 in R.

(12) Hi-C seq and MethylC seq analyses: We used HiC-Pro (v2.11.1) for mapping and calculating interaction matrix. HiC-seq connection heatmap was generated using HiCPlotter (https://github.com/kcakdemir/HiCPlotter). For MethylC seq, we used Bismark (v0.18.1) for mapping and methylKit (v1.2.4) to count methylated and unmethylated cytosines. We used python (v2.7.15) for comparing average HiC-seq statistics (number of connections, intensity or interaction matrix, and distance) and DNA methylation in each recombination spots. We used prcomp function in R (v3.5.1) to calculate correlation (r or r-square values).

(13) Genotyping, haplotype and recombination rate analyses: We used BLASTn (v2.7.1+) for SNP sequence alignment and Beagle (v4.1) and PLINK (v1.90b3.45) for SNP processing. PLINK (v1.90b3.45) and HaploView (v4.2) were used for haplotype block partitioning. The statistical programing language R (v3.5.2) was used for recombination rate analysis and graphical illustrations using the R packages "MareyMap" (v1.3.4) and "ggplot2" (v3.1.0), respectively.

For manuscripts utilizing custom algorithms or software that are central to the research but not yet described in published literature, software must be made available to editors/reviewers. We strongly encourage code deposition in a community repository (e.g. GitHub). See the Nature Research guidelines for submitting code & software for further information.

## Data

Policy information about availability of data

All manuscripts must include a data availability statement. This statement should provide the following information, where applicable:

- Accession codes, unique identifiers, or web links for publicly available datasets
- A list of figures that have associated raw data
- A description of any restrictions on data availability

SUBID  BioProject   BioSample   Accession   Organism
————————————————————————————
SUB5895679 PRJNA516411  SAMN10992405 VKDL00000000  Gossypium barbadense
SUB5895750 PRJNA516409 SAMN10884649 VKGI00000000 Gossypium darwinii
SUB5899309 PRJNA515894 SAMN11351207 VKGJ00000000 Gossypium hirsutum
SUB5899582 PRJNA516412 SAMN11289623 VKGE00000000 Gossypium tomentosum
SUB5901069 PRJNA525892 SAMN11110849 VKGF00000000 Gossypium mustelinum
Note: Assemblies are still in manual review and will be released under those accession numbers.
All other datasets were deposited in GenBank or GEO with accession numbers or shown in Supplemental Datasets or Tables.

# Field-specific reporting

Please select the one below that is the best fit for your research. If you are not sure, read the appropriate sections before making your selection.

☒ Life sciences ☐ Behavioural & social sciences ☐ Ecological, evolutionary & environmental sciences

For a reference copy of the document with all sections, see nature.com/documents/nr-reporting-summary-flat.pdf

# Life sciences study design

All studies must disclose on these points even when the disclosure is negative.

| | |
|---|---|
| Sample size | Sample size per group or condition was determined based on the minimum number of biological replicates required to perform differential expression analysis as per software tools used and previously published literature.<br><br>Sample size for linkage map analysis was determined based on the minimum number of individuals required to generate a linkage map. The number of cultivars included in the diversity panel was based on data availability and analytical sufficiency. |
| Data exclusions | Samples were excluded if they failed at the library preparation stage or those that displayed poor correlation between biological replicates.<br><br>SNPs were excluded if they did not meet the minimum BLASTn parameters for sequence alignment. A SNP was excluded if there was mapping ambiguity between the reference genome and the linkage mapping populations. This was done to reduce the occurrence of erroneous alignments that may result due to repetitive and homeologous sequences within the JGI G. hirsutum v2 reference genome. |
| Replication | Findings were consistent between biological replicates and different sequencing plates/batches.<br>Linkage mapping populations were not replicated due to resource constraints. |
| Randomization | Order of sample processing for library preparation and sequencing were processed in multiple batches as and when they were received from collaborating laboratories, kind of randomization in itself, but following stringent standardized protocols.<br><br>Linkage mapping software randomizes starting order of SNP markers across multiple iterations to determine optimal starting order. Randomization does not affect haplotype partitioning and thus was not used in the cultivar analysis. |
| Blinding | No blinding took place. To alleviate any complications from non-blinded analyses all samples were analyzed simultaneously in the same manner regardless of their condition/origin.<br>All specimens' identities were encoded before submission for genotyping. |

# Reporting for specific materials, systems and methods

We require information from authors about some types of materials, experimental systems and methods used in many studies. Here, indicate whether each material, system or method listed is relevant to your study. If you are not sure if a list item applies to your research, read the appropriate section before selecting a response.

## Materials & experimental systems

| n/a | Involved in the study |
|---|---|
| ☐ | ☒ Antibodies |
| ☒ | ☐ Eukaryotic cell lines |
| ☒ | ☐ Palaeontology |
| ☒ | ☐ Animals and other organisms |
| ☒ | ☐ Human research participants |
| ☒ | ☐ Clinical data |

## Methods

| n/a | Involved in the study |
|---|---|
| ☒ | ☐ ChIP-seq |
| ☒ | ☐ Flow cytometry |
| ☒ | ☐ MRI-based neuroimaging |

## Antibodies

| | |
|---|---|
| Antibodies used | Affinity purified anti-m6A rabbit polyclonal antibody (Synaptic Systems, cat. no. 202 003) |
| Validation | Information of Affinity purified anti-m6A rabbit polyclonal antibody (https://www.sysy.com/factsheets/202_003.pdf). |
